# Molecular Imprinting of Benzylpiperazine: A Comparison of the Self-Assembly and Semi-Covalent Approaches

**DOI:** 10.3390/ijms24065117

**Published:** 2023-03-07

**Authors:** Kathleen M. Wright, Michael C. Bowyer, Adam McCluskey, Clovia I. Holdsworth

**Affiliations:** 1 Sanitarium Health Food Company, 1 Sanitarium Drive, Berkeley Vale, NSW 2261, Australia; 2 School of Environmental and Life Sciences, University of Newcastle, Callaghan, NSW 2308, Australia; 3 Academic Division, University of Newcastle, Ourimbah, NSW 2258, Australia

**Keywords:** molecular imprinting, molecularly imprinted polymers, MIP, self-assembly MIP, semi-covalent MIP, benzylpiperazine, benzylpiperazine MIP

## Abstract

Molecularly imprinted polymers (MIPs) for benzylpiperazine (BZP, **1**), an illicit designer drug, were developed by using both self-assembly and semi-covalent approaches. From an array of potential functional monomers (FMs) and using a combination of pre-synthetic interaction studies (by molecular modelling and NMR analysis) and binding assays, the highest performing self-assembly **1**-MIPs were confirmed to result from methacrylic acid (**7**) as FM, ethylene glycol dimethacrylate (EGDMA) or trimethylolpropane trimethacrylate (TRIM) as crosslinkers and chloroform as the porogen and rebinding solvent at template (T): FM ratios of 1:1 and 1:2, giving imprinting factors (IF) 3 to 7. The semi-covalent **1**-MIPs were designed using benzylpiperazine (4-vinylphenyl) carbamate (**16**) as the template–monomer adduct in combination with either EDGMA or TRIM. Our comparative analysis showed the semi-covalent polymers to have a stronger affinity for **1** (significantly lower K_d_ values and higher IFs) and faster uptake than the self-assembly systems. Both approaches have comparable cross-reactivity: marginal to low against cocaine (**17**) and morphine (**18**) and high against ephedrine (**19**) and phenylpiperazine (**20**). They also have comparable selectivity: highly selective towards **1** against **17**, moderate against **18** and non-selective against **19**. EGDMA-based self-assembly MIPs displayed a greater imprinting effect (higher IFs and NIP-to-MIP K_d_ ratios) than TRIM-based MIPs, while the TRIM-based semi-covalent MIP outperformed its EGDMA-based equivalent. By virtue of its modest selectivity against the test illicit drugs, **1**-MIPs could potentially be used as a dummy MIP for the broad-based capture and enrichment of illicit drug blends for subsequent laboratory analysis.

## 1. Introduction

**Figure 1:**
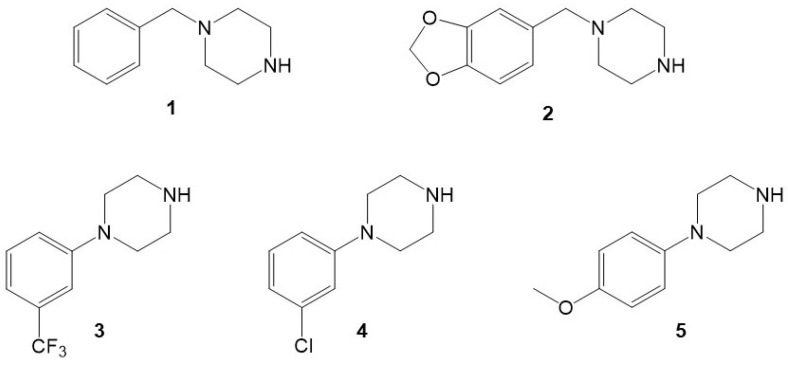
Chemical structures of benzylpiperazine (**1**) and its related analogues 1-(3,4-methylenedioxyphenyl)piperazine (**2**), 1-(3-trifluoromethylphenyl)piperazine (**3**), 1-(3-chlorophenyl)piperazine (**4**) and 1-(4-methoxyphenyl)piperazine (**5**).

Benzylpiperazine (BZP, **1**) is one of the dominant bioactive compounds in a relatively new class of piperazine-based illicit designer drugs in circulation [1]. This compound family includes 1-(3,4-methylenedioxyphenyl)piperazine (**2**), 1-(3-trifluoro-methylphenyl)piperazine (**3**), 1-(3-chlorophenyl)piperazine (**4**) and 1-(4-methoxyphenyl)piperazine (**5**) (Figure 1). The **1**-hydrochloride salt (its most widely distributed form) is a white powder that is usually sold as tablets or capsules. It acts as a stimulant, increasing blood pressure, auditory vigilance and heart rate [2]. The biological activity of **1** is thought to be mediated through 5-HT-uptake inhibition and 5-HT1 antagonism effects [2,3,4,5]. Co-administration of **1** with **3** results in psychoactive effects, including hallucinations, similar to those associated with 3,4-methylenedioxymethamphetamine (MDMA), and is 10 times more active than amphetamine [6,7]. Ingestion of **1** can have lethal outcomes [8,9]. Increasingly, countries have listed **1** as an illicit substance, but up until 2008, **1** was legally available to people over the age of 18 in United Kingdom and New Zealand [10,11].

A laboratory-based analyses of **1** could easily be conducted by using spectroscopic and chromatographic techniques [12,13], but reports on potential on-site detection have been limited to electrochemical methods [14,15] and presumptive colour tests [12,16]. While presumptive colour tests are rapid and cheap, these are prone to false positives, some tests are pH dependent and reagents have limited stability even with low temperature storage. Electrochemical methods rely on nonportable instrumentation with lower resolution than the chromatographic techniques. Spectroscopic on-site analysis is now possible with the emergence of portable instruments, e.g., FTIR; however, this would still require specialised training, as with all other forms of laboratory-based analytical instrumentation.

Molecular imprinted polymers (MIPs) have been used by our group [17,18] and others [19] for the capture and detection of illicit drugs. Nevertheless, there has been no report in the literature on MIPs designed for the capture of **1** to date. Thus, in this study, we aimed to develop **1**-selective MIPs, initially as an extraction and enrichment material and potentially as an on-site detection system. MIPs are generated from a functional monomer (FM), an analyte used as a template (T)—**1** in this study, a crosslinker (XL) for structural rigidity and a porogen (solvent) usually by radical polymerisation. Post-synthesis template extraction develops a complementary binding site within the polymer matrix, allowing highly selective analyte [20] or related analogue rebinding, i.e., dummy templating [21]. Due to their ease of synthesis, high stability and low cost, the use of MIPs as detection elements for several sensing platforms [22,23,24,25] is gaining momentum. At their simplest, MIPs are used as highly specific solid-phase extraction systems and afford easy identification and quantification by traditional analytical means [26,27,28,29,30,31].

**Figure 2:**
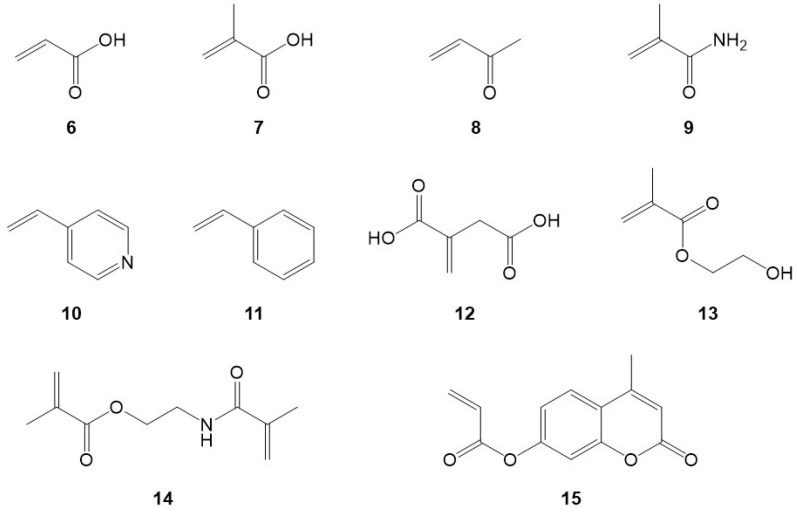
Chemical structures of functional monomers examined in this study: acrylic acid (**6**), methacrylic acid (**7**), acrylamide (**8**), methacrylamide (**9**), 4-vinylpyridine (**10**), styrene (**11**), itaconic acid (**12**), 2-hydroxyethyl methacrylate (**13**), *N,O*-bismethacroyloyl ethanolamine (NOBE) (**14**) and 7-hydroxy-4-methylcoumarin acrylate (**15**).

Here we report on our efforts towards the development of a **1**-selective MIP via self-assembly and semi-covalent approaches. Being synthetically simpler, a majority of MIPs fall into the former classification. With self-assembly MIPs, the FM, T, XL and porogen are pre-mixed and allowed to self-associate prior to polymerisation. To optimise the formulation, pre-synthetic approaches such as virtual imprinting [32,33,34], semi-empirical calculations [11], thermodynamic studies [35,36], spectroscopic (NMR [37,38], UV–VIS [39,40] and FTIR [41]) analyses, chemometric methods [42] and combinatorial screening [33,43,44] have been extensively employed. Commercially available FMs are commonly utilised, except in cases where specialty monomers are required. Covalent and semi-covalent MIPs, on the other hand, require the formation a T-FM adduct, which, at times, involves numerous synthetic steps. Regardless, the covalent (T attached covalently to FM moiety during binding) and semi-covalent (T-FM interaction during rebinding is non-covalent, as with the self-assembly systems) approaches have been highly successful, especially in cases where the parent template was poorly functionalised and thus offered limited possibilities for strong non-covalent interactions at the pre-polymerisation stage of MIP synthesis [45,46,47]. Our self-assembly MIP development process commenced with molecular modelling interaction and NMR titration (MM-NMR) studies to screen potential FMs and XLs (Figure 2) and determine the optimal FM-T interactions [17,18]. In the case of the semi-covalent approach, we synthesised and employed *O*-4-vinyl 4-benzylpiperazine-1-carbothioate (benzylpiperazine (4-vinylphenyl) carbamate, **16**, Figure 5) as a covalent surrogate for **1**.

We successfully prepared high-performing self-assembly **1**-MIPs from methacrylic acid (**7**, Figure 2) as an FM, at T:FM ratios of 1:1 and 1:2, using chloroform as a porogen (IF’s 3 to 7), with the EGDMA-based MIPs outperforming the TRIM MIPs. Nevertheless, the semi-covalent MIPs, particularly the TRIM-based polymers, have a stronger affinity for **1** (significantly lower K_d_ values and higher IFs) and faster uptake than the self-assembly systems. Both approaches exhibited comparable cross-reactivity—marginal to low against cocaine (**17**) and morphine (**18**) and high against ephedrine (**19**) and phenylpiperazine (**20**); and selectivity—highly selective towards **1** against **17**, moderate against **18** and non-selective against **19** (see Figure 7 for structures of test illicit substances).

An initial analysis might suggest that these high levels of cross-reactivity and modest selectivity against the tested illicit drugs were unfavourable; however, these **1**-templated MIPs are useful for broad-based capture and enrichment of illicit substances usually blended with, rather than specifically for, **1**, analogous to dummy-templated MIPs.

## 2. Results

### 2.1. The Self-Assembly Approach

#### 2.1.1. Template–Monomer Interaction Studies

Molecular modelling (MM) analysis (Supplementary Figures S1–S5) identified potentially favourable hydrogen bond interactions (<2.8 Å) [48] between **1**, by virtue of the nitrogens of the piperazine ring, and the FMs **6**, **7** and **12**–**15** (see Figure 2 for FM structures) at **1**-FM ratios <1:2. Subsequent **1**-FM NMR interaction studies, i.e., ^13^C NMR titration and Job plots (Supplementary Figures S9–S15), showed minimal or unfavourable interaction with **13**–**15**, but they did confirm favourable interactions with **6**, **7** and **12** at **1**-FM ratios <1:2. These interactions were demonstrated by significant upfield shifts in the resonance signals (2.5–6 ppm) of the carbons in the piperazine ring of **1** in the presence of FMs **6**, **7** and **12**. The resonance shifts could be attributed to the hydrogen-bond interactions between the adjacent nitrogens and the acidic moieties of the FMs. Conversely, the carbonyl carbons of FMs **6**, **7** and **12** also exhibited a downfield movement (deshielding) of their signals, an expected effect of the hydrogen-bond interactions. The Job plots generated for **1** and FMs **6**, **7** and **12** also showed well-defined 1:1 stoichiometries (Supplementary Figures S9–S11), further confirming favourable **1**-FM interactions.

Although some interaction between **1** and our selected XL agents, ethylene glycol dimethylacrylate (EGDMA), trimethylolpropane trimethylacrylate (TRIM) and divinylbenzene (DVB), were noted from computer simulations (Supplementary Figures S6–S8), ^13^C-NMR titration experiments presented very minimal **1**-XL interactions (Supplementary Figures S14 and S15), making them ideal for promoting **1**-FM association and the formation of template imprints.

#### 2.1.2. Selection of Crosslinker

XL makes up about 80% of a MIP formulation and potentially has the greatest influence on non-specific binding in MIPs. Thus, to further our molecular modelling and NMR studies, the affinity of **1** to EGDMA, TRIM and DVB polymers was measured. These crosslinked polymers were prepared in the absence of **1** and any FM in acetonitrile (AN) and CHCl_3_, the chosen porogens for **1**-MIPs. Although **14** has been successfully used both as a functional monomer and crosslinker in OmniMIPs [49] and our MM and NMR data suggest that it could potentially work as a crosslinker (though not as a functional monomer) for **1**-MIP, we excluded **14** in this study. High levels of non-specific binding of **1** were observed in DVB polymers, two to four times higher than in EDGMA and TRIM polymers. Of the three crosslinkers, only DVB is capable of π-π stacking interaction with **1**, and this is most likely the reason for the observed high level of superficial binding of **1**.

#### 2.1.3. Preparation of **1**-MIPs

Having identified **6**, **7** and **12** as suitable FMs from MM and NMR studies, the corresponding **1**-MIPs were prepared in T:FM ratios of 1:1, 1:2 and 1:4 in CH_3_CN and CHCl_3_ as porogens and EGDMA and TRIM as crosslinkers. The T:FM ratios of 1:1 and 1:2 were chosen as per our MM and NMR results, showing the most favourable interaction between **1** and FMs **6**, **7** and **12** at these ratios. The 1:4 T:FM ratio is the most common ratio used in the literature to promote T-FM interactions and complex formation. The **1**-MIPs were prepared in acetonitrile and chloroform to determine the effect of porogen polarity on the binding capabilities of the MIPs. Similarly, the influence of the level of crosslinking on the binding efficiency of **1**-MIPs was also evaluated by employing di- (EGDMA) and tri-(TRIM) XLs.

All 36 formulations of **1**-MIPs were prepared using 7 mL of porogen (~1 mmol FM + XL per 1 mL), with AIBN as the initiator, at 60 °C for 12 h. The resulting MIPs were ground with the fraction 32–63 µm collected and Soxhlet extracted with 10% acetic acid in methanol, followed by pure methanol. Extraction was repeated until **1** could no longer be detected in the HPLC trace of the methanol extract.

Subsequent reference to these **1**-MIP formulations follows the following codes: E and T for EGDMA and TRIM XLs, respectively; **6**, **7** and **12** for the corresponding FM, 1, 2 or 4 for 1:1, 1:2 and 1:4 T:FM ratios, respectively; and CH_3_CN or CHCl_3_ for the porogens. Thus, E**7**_1-MIPCHCl3_ refers to a **1**-MIP prepared using EGDMA as XL and **7** as FM in a 1:1 T:FM ratio, with chloroform as the porogen.

#### 2.1.4. Physical Characterisation

**Figure 3:**
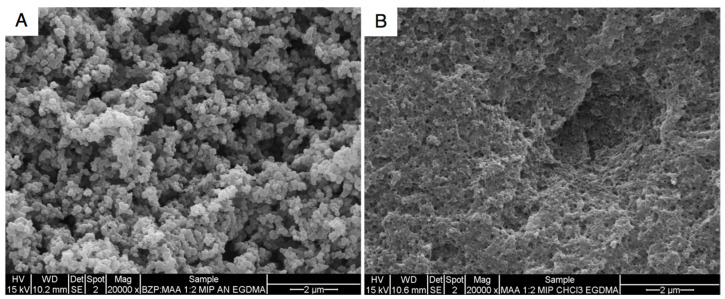
SEM images for (**A**) E**7**_2_-MIP_CH3CN_ and (**B**) E**7**_2_-MIP_CHCl3_ obtained at 20,000× magnification, at 15.0 kV.

Scanning electron microscopy was used to examine the surface morphology of both MIPs and NIPs (non-imprinted polymer). No significant difference in surface morphology was observed between MIPs and their corresponding NIPs. MIP surface morphology was unaffected by variations in the FM or T:FM ratio, but differences based on XL were apparent (Supplementary Figures S16 and S17). The TRIM-based MIPs appeared to be more compact, more dense and less porous than the surface of the EGDMA crosslinked polymers (Supplementary Figure S18). This was consistent with previous reports, which indicate that the nature of the crosslinker can affect polymer surface morphology [50]. It is also clear from Figure 3 that using CH_3_CN as the porogen produced a macroporous surface morphology, while CHCl_3_ produced smoother, more dense surfaces with fewer visible pores. This most likely resulted from a delayed phase separation from CHCl_3_; that is, the growing polymer was more soluble in CHCl_3_ than in CH_3_CN, a product of solvent–polymer polarity mismatch [51]. The porogen effect on morphology was evident in swelling experiments, wherein all CHCl_3_ polymers displayed an enhanced swelling capacity relative to the equivalent CH_3_CN polymers.

#### 2.1.5. Evaluation of the Imprinting Effect

The rebinding of **1** was initially evaluated by batch adsorption experiments, using 0.8 mM solution of **1** in CH_3_CN or CHCl_3_ with 30 min exposure to polymers of various masses (5, 10, 20 and 30 mg). As expected, the rebinding capacity was a function of the polymer mass used, typically plateauing at 20–30 mg polymer loading and increasing with increasing proportion of FM for both MIPs and NIPs. Our data suggest that the rebinding of **1** was not affected by the nature of the crosslinker; instead, it was porogen and FM dependent. In all instances, higher rebinding was observed with the CH_3_CN MIPs, but the imprinting effect was enhanced in CHCl_3_ MIPs, as a consequence of lower NIP rebinding of **1**.

**Figure 4:**
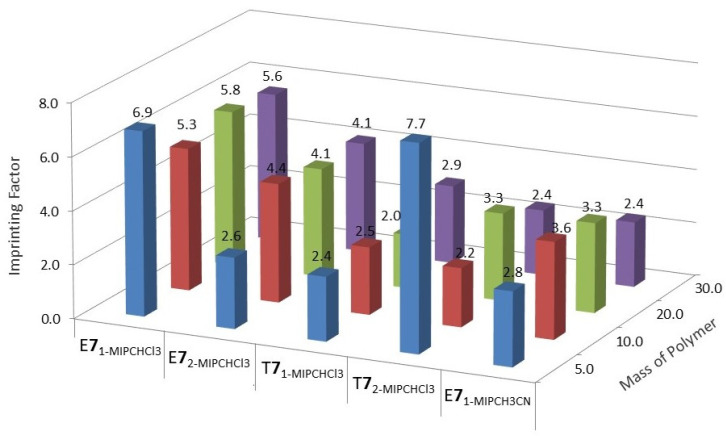
Imprinting factors (IFs) for the better-performing **1**-MIPs (≥2) from the set of formulations utilising **6, 7** and **12** as FMs, EGDMA and TRIM as XLs and CH_3_CN and CHCl_3_ as porogens. Rebinding studies were conducted using 1 mL of 0.8 mM solution of benzylpiperazine in the original porogen, with the uptake measured after 30 min. All binding tests were performed in triplicate.

This initial rebinding assays allowed us to screen MIPs and select those with an imprinting efficacy or imprinting factor IF (MIP binding/NIP binding) of ≥2 for all mass loadings for further binding characterisation. This imprinting efficacy criterion was obtained with MIPs E**7**_1-MIPCHCl3_, E**7**_2-MIPCHCl3_, T**7**_1-MIPCHCl3_ and T**7**_2-MIPCHCl3_, with CHCl_3_ as binding solvent; and E**7**_1-CH3CN_, bound in CH_3_CN (Figure 4). Template uptake by these polymers varied from 17 to 75% and from 3 to 26% across the MIPs and their corresponding NIPs, respectively.

#### 2.1.6. Binding Isotherms

The minimum time required to reach optimal template binding for MIPs E**7**_1-MIPCHCl3_, E**7**_2-MIPCHCl3_, T**7**_1-MIPCHCl3_ and T**7**_2-MIPCHCl3_ was determined prior to undertaking any other binding assays. No marked difference in the rebinding of **1** was observed for incubation times between 30 min and 24 h. Thus, we opted to use 60 min for subsequent binding tests. As only one MIP from the CH_3_CN series (E**7**_1-CH3CN_) exhibited noteworthy imprinting, no further analysis of this series was undertaken.

The binding isotherms were generated by varying the rebinding solution concentration of **1** between 0.5 and 10 mM, using a constant polymer loading of 20 mg and 60 min incubation (Supplementary Figure S19A–D). Maximum binding (B_max_) and affinity constants (K_d_) were calculated from non-linear regression of the Langmuir binding isotherms, using GraphPad Prism 9.4.1; the best-fit values obtained from the one-site–total-binding equation are presented in Table 1. These data show MIPs bound twice the amount of **1** than their corresponding NIPs, and this is attributable to the imprinting effect. E**7**_2_ and T**7**_2_ polymers (MIPs and NIPs) bound twice the amount of **1** than E**7**_1_ and T**7**_1_ polymers, a result that is unsurprising given that the E**7**_2_ and T**7**_2_ feed formulations contain twice the amount of functional monomer **7**.

The K_d_ values for the MIPs (0.22–0.62 µM) were consistently lower than the corresponding NIPs (0.71–1.52 µM) because of the higher affinity template binding arising from the imprinting process. Furthermore, the K_d_ values were higher for the 1:1 (E**7**_1_ and T**7**_1_) than for the 1:2 (E**7**_2_ and T**7**_2_) formulations, suggesting that the affinity for **1** was stronger at a higher functional monomer content. The NIP-to-MIP K_d_ ratios were higher for the EGDMA-based polymers (~5) than for the TRIM-based polymers (~2) and were consistent with the rebinding results and imprinting factors presented in Figure 4.

### 2.2. The Semi-Covalent Approach

**Figure 5:**
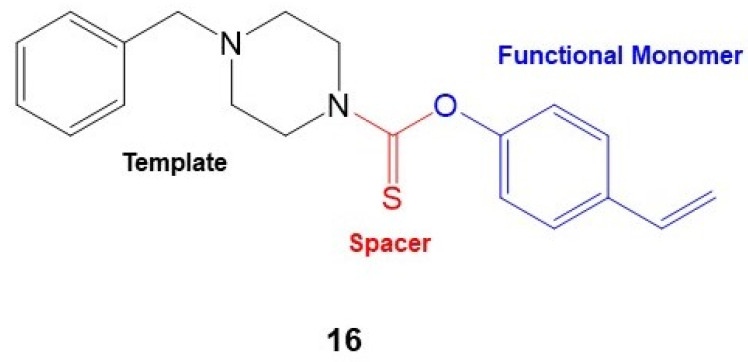
Chemical structure of *O*-4-vinyl 4-benzylpiperazine-1-carbothioate or benzylpiperazine (4-vinylphenyl) carbamate (**16**).

**Scheme 1:**
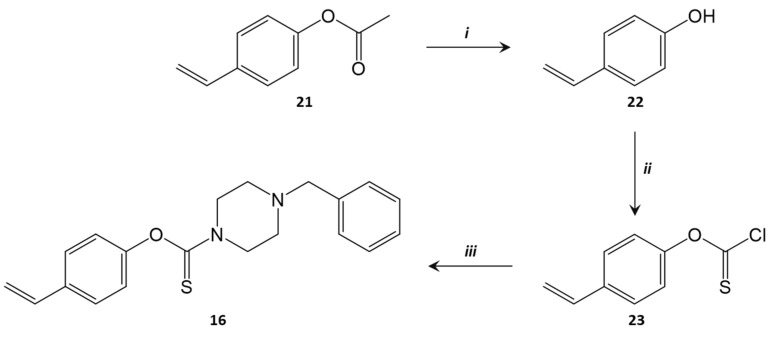
Reagents and conditions: (i) KOH and CO_2_ (g), 0–5 °C; (ii) NaOH, thiophosgene (SCCl_2_) and CHCl_3_, 0 °C; and (iii) NaOH, 0 °C.

The semi-covalent imprinting of **1** was undertaken by using *O*-4-vinyl 4-benzylpiperazine-1-carbothioate or benzylpiperazine (4-vinylphenyl) carbamate (**16**) as the template–monomer (TM) adduct (Figure 5 and Scheme 1). Post-polymerisation cleavage of the carbothioate linker would yield a proximally spaced phenol moiety capable of rebinding to **1** through hydrogen bonding upon re-exposure. The 4-vinylphenol moiety was previously utilised in the imprinting of cholesterol [52], N-heterocycles [53], profenofos and carbfuran [54] and substituted phenols [55].

Having synthesized the TM adduct **16**, MIPs were prepared in a 1:19 **16**:XL (EGDMA and TRIM) ratio in chloroform to give E**16**_MIPCHCl3_ and T**16**_MIPCHCl3_, respectively. The corresponding NIPs were prepared under identical conditions, using the XLs only.

SEM micrographs of the MIPs displayed clear evidence of a macroporous surface structure (Supplementary Figure S20) consistent with the surface structure expected from low-polarity solvents such as CHCl_3_ [18,51,56,57,58,59]. Both E**16**_MIPCHCl3_ and T**16**_MIPCHCl3_ exhibited low levels of swelling, with volume increases of 3–4% only upon exposure to the porogen CHCl_3_.

#### 2.2.1. Evaluation of Imprinting Effect

**Figure 6:**
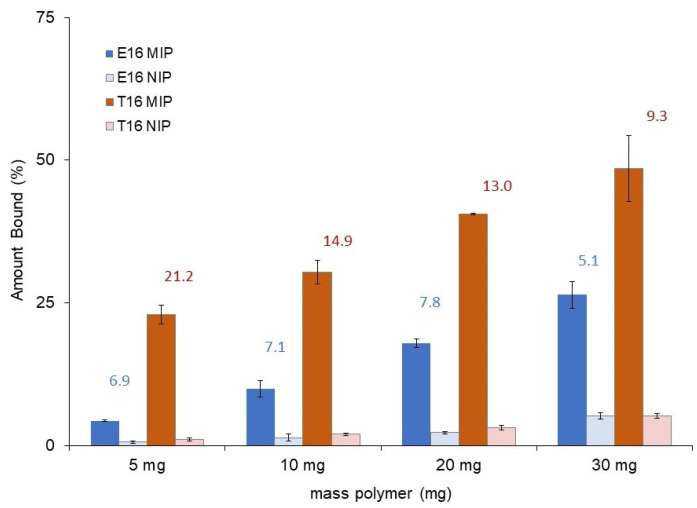
Rebinding results for E**16**_MIPCHCl3_ and T**16**_MIPCHCl3_ in CHCl_3_, using 1 mL 0.8 mM solution of **1** with a binding time of 30 min. All experiments were performed in triplicate. Numerical values above the bars indicate imprinting factor (IF) values.

E**16**_MIPCHCl3_ and T**16**_MIPCHCl3_ were evaluated for their ability to selectively rebind **1** by batch adsorption experiments, using 1 mL of 0.8 mM solution of **1** in CHCl_3_ and an incubation time of 30 min (as with the self-assembly MIPs). Both MIPs displayed a steady increase in template rebinding as a function of increasing polymer loading (from 5 mg to 30 mg), as is consistent with binding-site homogeneity [60]. The NIPs, on the other hand, displayed minimal binding, and this only slightly increased across all polymer loadings evaluated (Figure 6).

T**16**_MIPCHCl3_ showed a greater affinity for **1**, binding at least twice the amount absorbed by E**16**_MIPCHCl3_, with maximum binding at 30 mg polymer loading, resulting in 49% and 26% uptake of **1**, respectively. This result is in keeping with previous reports suggesting that XLs with more than two polymerizable groups, such as TRIM, result in a more porous macrostructure with improved mass transfer properties (better access to the imprinted cavities) and higher capacities [61,62].

The imprinting effect, measured by the imprinting factor (IF; amount bound by MIP/amount bound NIP) ranged from 5.1 to 21, results consistent with the presence of well-defined **1**-specific cavities (Figure 6). It should be noted, however, that the NIPs lacked the FM component usually associated with NIP synthesis, as such, producing a poorer mimic on a non-imprinting effect, but nonetheless one that has been used in semi-covalent imprinting previously [52,63].

#### 2.2.2. Binding Isotherms

Uptake of **1** was observed to be rapid, with optimal binding noted after 10 min for both MIPs. Binding isotherms (Supplementary Figure S19E) were generated across a 0.3–10 mM concentration range (of **1**) in CHCl_3_, using 30 mg of polymer and a 60 min rebinding time in order to be consistent with the self-assembly systems. The binding constant, K_d_, and B_max_ were calculated using non-linear regression of the Langmuir binding isotherms generated from GraphPad Prism, with the best-fit values obtained from the one-site–total-binding equation presented in Table 1. Since the NIPs showed negligible binding of **1**, only the MIPs were analysed. The K_d_ value for T**16**_MIPCHCl3_ (0.02 μM) is 5 times lower than E**16**_MIPCHCl3_ (0.09 μM), as is consistent with earlier observations (Figure 6) that the former has a greater affinity for **1**. These K_d_ values are also 3 and 33 times lower than the equivalent self-assembly MIPs (E7_1-MIPCHCl3_ and T7_1-MIPCHCl3_), respectively. The binding capacities (B_max_) of these semi-covalent MIPs are close in values and comparable to T7_1-MIPCHCl3_ but 3 times lower than E7_1-MIPCHCl3_.

### 2.3. Selectivity Studies

**Figure 7:**
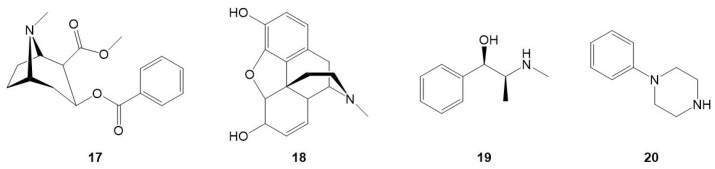
Chemical structures of illicit drugs used in competition and selectivity studies for **1**-based MIPs: cocaine (**17**), morphine (**18**), ephedrine (**19**) and phenylpiperazine (**20**).

Both self-assembly (E**7**_1-MIPCHCl3_, E**7**_2-MIPCHCl3_, T**7**_1-MIPCHCl3_ and T**7**_2-MIPCHCl3_) and semi-covalent (E**16**_MIPCHCl3_ and T**16**_MIPCHCl3_) MIP systems were subjected to selectivity tests—single component (cross-reactivity) and binary competitive rebinding of **1** against cocaine (**17**), morphine (**18**), ephedrine (**19**) and phenylpiperazine (**20**) (Figure 7). These tests were conducted using 20 mg of polymer in 0.8 mM solutions of analyte in CHCl_3_, with an incubation time of 60 min. A range of illicit drugs and precursors containing similar functionalities were chosen as competing analytes to simulate in-field capability for illicit detection.

#### 2.3.1. Cross-Reactivity Tests

The results for the non-competitive cross-reactivity assays are given in Supplementary Figure S21, while Table 2 shows the cross-reactivity indices (XRF) to enable a direct comparison of the uptake of each competing analyte by the MIP with respect to **1**. The XRF for an analyte is defined as the ratio of the analyte (**17**, **18**, **19** or **20**) to **1** MIP binding within the MIP.

The four self-assembly MIPs (E**7**_1-MIPCHCl3_, E**7**_2-MIPCHCl3_, T**7**_1-MIPCHCl3_ and T**7**_2-MIPCHCl3_) displayed high levels of cross-reactivity with **19** and **20**, with the XRFs ranging from 0.82 to 1.44, low to moderate with **18** (XRF = 0.23 to 0.43) and negligible with **17** (XRF = 0.08 to 0.15). Selective binding (ΔB = MIP binding − NIP binding) followed the same trend. These results reflect the effect of functional group character (electronic, size), relative proximity and orientation on each analyte with respect to **1**. Analytes **17** and **18** are both large molecules that differ in shape, potential and available functional groups to **1,** leading to a poor ‘fit’ within the imprinted cavities (Supplementary Figure S22). Consequently, they displayed low sorption and low affinity to the MIPs. In contrast, **19** and **20** are similar in size and chemical character to **1**, consistent with the observed high XRFs (Supplementary Figure S23). The very high uptake of **19,** equivalent or higher than **1,** could be attributed to multipoint H-bonding interactions with the MAA FM by virtue of its NH and OH groups, which are H donor/acceptor species, leading to potentially greater binding affinity. Furthermore, analyte **19** is the only test target containing an amine unit that is not part of a ring system, which could mean greater fluxionality, resulting in better orientation within the binding cavity.

The semi-covalent MIPs (E**16**_MIPCHCl3_ and T**16**_MIPCHCl3_) showed a cross-reactivity trend that is similar to the self-assembly MIPs, except for analyte **17**, which recorded high levels of cross-reactivity (XRF’s 0.64 and 0.85 for E**16**_MIPCHCl3_ and T**16**_MIPCHCl3_, respectively) in contrast to its behaviour towards the self-assembly MIPs. Note that the post-polymerisation cleavage of the carbothioate linker of **16** (Figure 5) to release the template moiety leaves a phenol functionality within the cavity capable of interacting with **1** or other complementary analytes. The XRFs for **19** and **20** are slightly lower than those observed with the self-assembly MIPs, thus suggesting a less favourable affinity to the imprinted cavities containing the phenol functionality than with the MAA carboxyl unit. The unexpected high cross-reactivity with **17** could possibly be due to its benzoyl group, which is similar in structure and size to the benzyl group of **1**, that could easily fit the imprinted cavities and interact with the phenol group by pi–pi stacking.

#### 2.3.2. Binary Competitive Assays

The selectivity of the MIPs was further tested using binary mixtures of **1** and competing analytes **17**, **18** and **19**. Analyte **20** was not pursued in this assay due to HPLC separation issues with **1**.

The uptake of all analytes was observed to be higher for MIPs than in their corresponding NIPs (Supplementary Figure S24), thus suggesting that they have access to and an affinity for the imprinted cavities. To facilitate binding selectivity analysis, we introduced the quantitative values summarized in Table 3. First, the uptake of **1** as the sole analyte, a measure of the MIP binding capacity, is taken as a reference; the binding of binary analytes is then normalised against this reference. Thus, if the normalised binding of an analyte is greater than 1, i.e., greater than 100%, then this means that its uptake is higher than the expected binding capacity. The selectivity index (SI) of an analyte is defined here as the ratio of normalised analyte binding to normalised **1** binding.

Although selectivity assessment is more essential for MIPs than NIPs, and despite the fact that NIP binding could be variable due to its superficial nature, the results of this study showed analyte **19** to be competitive with **1** in all NIP systems, giving normalised binding values as high as 3.15. The binding of **17** and **18**, however, was always lower or equivalent to that of **1**. Uptake of **1** by the semi-covalent NIPs is lower than the reference in all cases, while self-assembly NIPs registered **1** binding higher than the reference in some binary mixtures.

In the case of MIPs, **19** was also observed to be highly competitive, reducing the uptake of **1** to a low of 0.42, i.e., 42% (**T16**_MIPCHCL3_), with respect to the reference and displayed normalised binding and SIs higher than **1** (as high as 1.73 and 1.85, respectively, with **E7**_1-MIPCHCL3_) in all cases. Analyte **18** displayed low levels of competition with **1** in the self-assembly MIPs (SI = 0.16 to 0.37), slightly reducing the binding of **1** in some cases. However, the presence of **18** brought about a significant reduction in **1** binding (below 50%), resulting in a moderate SI of 0.46 (**E16**_MIPCHCL3_) and 0.61 (**T16**_MIPCHCL3_) in the case of the semi-covalent MIPs. Analyte **17** did not have a significant impact on the binding of **1**, except in **T16**_MIPCHCL3_, and displayed low-to-minimal competition tendency against **1**, with normalized binding and SI values all being <0.2. In some instances (also observed with the NIPs), the presence of **17** and **18** enhanced the uptake of **1**, which could be attributed to its interaction with these analytes by hydrogen bonding and/or pi–pi stacking. On the other hand, in the presence of **19**, the uptake of **1** in all polymers tested was reduced, thus confirming its observed competitive tendency against **1**.

Consistent with the results of the non-competitive cross-reactivity assays, the results of the competitive (binary mixtures) binding studies showed all MIPs to be highly selective towards **1** in the presence of **17** (SI < 0.2), moderately selective in the presence of **18** (SI > 0.2 to ~0.6) and non-selective in the presence of **19** (SI > 1).

#### 2.3.3. Selectivity of **1**-MIPs: Implication on Their Applications

The poor-to-moderate selectivity of **1**-MIPs against **18**, **19** and **20** could initially be construed as unfavourable; however, these results indicate that **1**-MIPs are capable of broad-based capture of blends of illicit substances with, rather than specifically for, **1**, comparable to the ‘dummy’ MIP approach. The high cross-reactivity against **20** means that **1**-MIPs could potentially extract 1-(3-trifluoromethyl-phenyl)piperazine (TFMPP, **3**), a derivative of **20** most commonly blended with **1**, and other known substituted phenylpiperazine blend ingredients such as **4**, **5** (Figure 1) and 4-fluorophenylpiperazine [64]. Other pills are also known to be mixed with cocaine (**17**) [16,65] and ephedrine (**19**) [64], which, according to our selectivity studies, could also be extracted by **1**-MIPs.

Illicit drugs are never pure, and conducting an analysis of their composition, including minor ingredients and adulterants, is essential in drug profiling studies [66]. The competitive uptake of **18**, **19** and **20** by **1**-MIPs means that, when present in trace amounts, they could be pre-concentrated within the MIP, which could enhance their detection. We speculate that other minor non-piperazine derivatives blended with **1** could also be captured and pre-concentrated with **1**-MIPs. Unfortunately, due to the nature of these analytes, we could not easily obtain real samples to test.

Our results suggest no significant difference in the selectivity between self-assembly and semi-covalent **1**-MIPs. Thus, the MIP materials could be prepared by the synthetically simpler self-assembly imprinting using cheap commercially available monomers (monomer **7** and crosslinkers), thus making these materials very competitive over other analytical extraction methods.

Our **1**-MIP materials are suited to laboratory test setting, with potential as the recognition element for in-field sensing devices. Sample preparation would be simple, and with their enhanced enrichment capability, due to the presence of imprinted sites, they would be useful for the capture of illicit drugs, particularly in biological samples. MIP-bound drug analytes could be re-extracted and differentiated by a number of analytical procedures—notably, chromatographic methods—preferably with MS detection and capillary electrophoresis, among others.

## 3. Materials and Methods

### 3.1. Reagents

Benzylpiperazine (**1**) was purchased from Fluka and used as received. Azobisisobutyronitrile (AIBN) was obtained from DuPont and was recrystallised from acetone prior to use. Acrylic acid (**6**), methacrylic acid (**7**), itaconic acid (**12**), divinylbenzene (DVB), ethylene glycol dimethacrylate (EGDMA), trimethylolpropane trimethacrylate (TRIM), sodium hydroxide, glacial acetic acid, hydrochloric acid, potassium hydroxide, (1*R*, 2*S*)-(-)-ephedrine (**19**), 1-phenylpiperazine (**20**), p-acetoxystyrene (**21**), thiophosgene and 7-hydroxy-4-methylcoumarin were purchased from Sigma-Aldrich and used as received, unless otherwise stated. Monomers **6** and **7** and the crosslinkers DVB and EGDMA were distilled under reduced pressure prior to use. TRIM was purified by washing with aqueous sodium hydroxide (0.1 M, 2 × 50 mL), water (50 mL) and saturated brine solution (50 mL) and then dried over MgSO_4_. *N,O*-bismethacryloyl ethanolamine (NOBE, **14**), 7-hydroxy-4-methylcoumarin acrylate (**15**) and benzylpiperazine (4-vinylphenyl) carbamate (**16**) were synthesized in-house, according to the synthetic procedures outlined below. Cocaine base (**17**) and morphine (**18**) was provided by the Australian Federal Police Forensic Services and was used as received. Methacryloyl chloride and acryloyl chloride were purchased from Fluka and Lancaster, respectively, and used as received.

HPLC-grade acetonitrile and chloroform were obtained from Merck and were used as received. All other solvents were distilled prior to use, unless otherwise stated.

Deuterated chloroform (99.8%) and dimethylsulfoxide (99.8%) for NMR analysis were obtained from Cambridge Isotope Laboratories Incorporated.

### 3.2. Preparation of N,O-Bismethacryloyl Ethanolamine (NOBE, ***14***)

NOBE (**14**) was prepared as per the method utilised by Sibrian-Vazquez and Spivak [49]. Ethanolamine (0.976 g, 16 mmol) was mixed with 15 mL of dichloromethane. TEA (3.74 g, 5.15 mL, 37 mmol) was added in small portions to the initial mixture, with stirring, and the reaction mixture was cooled to 0 °C. Methacryloyl chloride (3.867 g, 3.6 mL, 37 mmol) was added dropwise, with vigorous stirring, while keeping the temperature at 0 °C. After the complete addition of methacryloyl chloride, the temperature was increased to 40 °C and allowed to react for 24 h at this temperature. The reaction mixture was filtered, and the precipitate (Et3NHCl) was then discarded. The filtrate was extracted with 0.5 M NaHCO3 (3 × 15 mL) and 0.5 M sodium citrate (3 × 15 mL). The solvent was evaporated under vacuum, the compound was isolated by column chromatography (EtOAc/hexanes 50:50, EtOAc 100%), and **14** was isolated as a pale yellow oil. Yield: 59%. ^1^H NMR (CDCl3, 300 MHz): δ/ppm = 6.80, 5.99, 5.71, 5.60, 5.38, 4.25, 3.58 1.97, 1.89. ^13^C NMR (CDCl3, 75.5 MHz): δ/ppm = 168.5, 167.4, 139.8, 135.9, 126.0, 119.4, 63.2, 39.0, 18.4, 18.1.

### 3.3. Preparation of 7-Hydroxy-4-methylcoumarin Acrylate (***15***)

First, **15** was prepared by the literature method [67]. TEA (12.1 g, 120 mmol) and 7-hydroxy-4-methylcoumarin (8.809 g, 50 mmol) were dissolved in chloroform (150 mL) and cooled to 0 °C. Acryloyl chloride (10.9 g, 120 mmol) was added dropwise, with vigorous stirring. The reaction mixture was then returned to room temperature and stirred for a further 12 h. The solvent was evaporated under vacuum, and the product was purified by dissolving the precipitate in methanol (200 mL). The methanol solution was then poured into water (1 L), the precipitate was collected by filtration, and **15** was isolated as a white flake. ^1^H NMR (CDCl3, 300 MHz): δ/ppm = 7.86, 7.39, 7.28, 6.67, 6.62, 6.52, 6.48, 6.24, 3.41. ^13^C NMR (CDCl3, 75.5 MHz): δ/ppm = 163.8, 159.7, 153.6, 153.0, 152.6, 134.4, 127.3, 126.5, 118.4, 117.7, 113.9, 110.2, 18.2.

### 3.4. Synthesis of Benzylpiperazine (4-Vinylphenyl) Carbamate (***16***)

#### 3.4.1. Synthesis of 4-Vinylphenol (***22***) [68]

4-Acetoxystyrene (**21**) (2.0 g, 13.22 mmol) was added to potassium hydroxide (2.0 g, 35.65 mmol) in water (25 mL) and stirred at 0–5 °C until homogeneous (5 h). Gaseous carbon dioxide was passed into the stirred cold solution to pH 8 to produce p-vinylphenol (**22**) [62]. Yield: 61%. ^1^H NMR (CDCl_3_, 300 MHz): δ 7.30, 6.79, 6.63, 5.54, 504. ^13^C NMR (CDCl_3_, 75.5 MHz): δ 158.4, 137.6, 130.3, 128.4, 116.0, 110.9.

#### 3.4.2. Synthesis of 4-Vinylphenyl Chlorothioformate (***23***) [69]

A solution of **22** (0.5 g, 4.2 mmol) in 5% NaOH (5 mL) was added dropwise, with stirring, to a solution of thiophosgene (0.45 g, 4.2 mmol) in chloroform (3 mL) cooled to 0 °C. The reaction was stirred for 1 h, at 0–5 °C, and the chloroform layer washed with dilute HCl and water. Solvent was removed under reduced pressure, and product **23** was separated by column chromatography (silica gel, 10% EtOAc:hexane). Yield: 85%. ^1^H NMR (CDCl_3_, 300 MHz): δ 7.46, 7.08, 6.67, 5.70, 5.26. ^13^C NMR (CDCl_3_, 75.5 MHz): δ 185.7, 154.0, 136.8, 135.4, 127.6, 121.2, 120.9, 115.2.

#### 3.4.3. Synthesis of *O*-4-Vinyl 4-Benzylpiperazine-1-carbothioate (**16**)

4-Vinylphenyl chlorothioformate (**23**) (0.7 g, 3.54 mmol) was added dropwise to **1** (1.25 g, 7.09 mmol), with stirring. The reaction was stirred for 1 h, and product **16** was separated by column chromatography (silica gel, 5% to 10% EtOAc:hexane). Yield: 40%. ^1^H NMR (CDCl_3_, 300 MHz): δ 7.30 (d), 7.21 (m), 6.89 (d), 6.58 (q), 5.60 (d), 5.13 (d), 4.09 (m), 3.88 (m), 3.50 (s), 2.51 (d) ppm. ^13^C NMR (CDCl_3_, 75.5 MHz): δ 186.9, 153.4, 136.1, 135.7, 129.5, 128.7, 127.8, 127.1, 123.0, 114.3, 62.8, 52.6, 52.4, 50.2, 46.3.

### 3.5. Molecular Modelling

Template–monomer molecular interactions were modelled using Spartan ’04 software, using the AM1 force field. This molecular orbital computational method predicts the stable configuration of the template (T), functional monomer (FM), FM-FM clusters and T-FM clusters and calculates their standard heats of formation (ΔH_f_). The molecules were randomly positioned, and the T-FM clusters were modelled with respect to increasing the template–monomer ratio from 1 to 4. To account for the FM-FM interaction, the FM-FM clusters of up to five molecules were also surveyed. The energies of interaction of the T-M clusters, ΔE° (cluster), at different molecular ratios were then calculated using the following equation:

ΔE_interaction_ = ΔH_f_ FM-T complex − [ΔH_f_ monomer cluster − ΔH_f_ template] (1)

### 3.6. NMR Spectroscopic Analysis

^1^H and ^13^C Nuclear Magnetic Resonance (NMR) spectra were recorded at 300.13 and 75.47 MHz, respectively, using a Brüker Advance 300 MHz Spectrometer in conjunction with Brüker Topspin v1.3 software. Experiments involving **6**, **7** and **14** were performed in CDCl_3_, while experiments with **12** were performed in DMSO-*d_6_* at a temperature of 301 K. For the NMR titration, molar aliquots of monomer were sequentially added to the template **1** (0.1 mmol) up to a maximum of 16 equivalents. After each aliquot addition, the sample was mixed and allowed to spin for five minutes before spectrum acquisition. The experiment was repeated in the absence of **1**. For the Job experiments, 11 samples were prepared with varying **1** and monomer molar ratios, ranging from 0 to 1, using 0.2 mM solutions. The total volume was constant at 0.5 mL.

### 3.7. Polymer Synthesis and Template Extraction

#### 3.7.1. Self-Assembly MIPs

The required amounts of functional monomer (0.34 mmol, 0.68 mmol or 1.36 mmol) and crosslinker (6.64 mmol, equivalent to 20 × T) were added to a solution of **1** (60 mg, 0.34 mmol) in 7 mL porogen (CH_3_CN or CHCl_3_). Based on our MM-NMR analysis, **6**, **7** and **12** were selected as functional monomers and EGDMA and TRIM as crosslinkers. MIPs were prepared using 1:1, 1:2 and 1:4 T:M ratios in chloroform and acetonitrile. The reaction mixture was degassed with N_2_ before AIBN (50 mg) was added. The mixture was heated to 60 °C in an oven (Thermoline). NIPs were prepared by using the same method but without the addition of **1**.

Polymers were ground wet in methanol and sieved with the fraction between 32 and 65 μm collected. Template removal was by Soxhlet extraction, using a 10% acetic acid-methanol mix for 48 h, followed by 100% methanol for 12 h. The polymers were dried at 40 °C for 24 h.

#### 3.7.2. Semi-Covalent MIPs

Benzylpiperazine(4-vinylphenyl)thiocarbamate (**16**) (115 mg, 0.34 mmol) was mixed with crosslinker (EGDMA or TRIM, 6.46 mmol) and AIBN (1% mol ratio) in chloroform (2 mL/g monomers). The reaction mixture was degassed with N_2_ and then heated to 60 °C in an oven (Thermoline). NIPs were prepared using the same method but without the addition of **16**.

Polymers were ground wet in methanol and sieved with the fraction between 32 and 65 μm collected. The template adduct was cleaved by heating the polymer at reflux over 1 M NaOH for 12 h and then neutralised with dilute HCl. The polymers were then washed with methanol for 12 h, using a Soxhlet extractor. Finally, the polymers were dried at 40 °C for 24 h [48,49,55].

### 3.8. Batch-Binding Tests for ***1***

#### 3.8.1. HPLC Analytical Method

Batch rebinding experiments were carried out using a known concentration of **1** stock solution in either acetonitrile or chloroform. The required mass of polymer was left in contact with the **1** solution for the required time. The quantification of **1** was achieved by HPLC, using a Shimadzu High Performance Liquid Chromatograph (HPLC) (LC-20AD) fitted with an EconosphereTM C18, 5 μm column (Grace^®^).

For **1** binding in CH_3_CN, the mobile phase comprised 50% CH_3_CN and 50% buffer solution (25 mM K_2_HPO_4_; 30 mM KCl; 7 mM TEA; adjusted to pH 3 with H_3_PO_4_). A 10 μL injection volume was used with a run time of 10 min, flow rate of 2 mL min^−1^ and detection wavelength of 254 nm.

For binding in CHCl_3_, the mobile phase consisted of 70% CH_3_CN and 30% buffer solution (25 mM K_2_HPO_4_; 30 mM KCl; 7 mM TEA; adjusted to pH3 with H_3_PO_4_). A 10 μL injection volume was used with a run time of 15 min, flow rate of 0.95 mL.min-1 and detection wavelength of 254 nm. A calibration curve was generated using six solutions in the range of 0.1 to 0.8 mM.

#### 3.8.2. Sorption Tests: Evaluation of Imprinting Effect

A sorption study to evaluate the imprinting efficiency was performed. Various polymer masses from 10.0 to 30.0 mg were placed into 5 mL vials to which was added 1.00 mL of 0.0800 mM **1** in CH_3_CN or chloroform. The mixture was shaken for 30 min, filtered and the filtrate analysed directly by HPLC. The amount of free **1** was subtracted from the initial binding solution concentration to obtain the amount of 1 bound in the polymer. All binding experiments for this study were performed in triplicate to ensure reproducibility.

Results of the sorption tests were favourable for only 4 of the self-assembly systems tested: E**7**_1-MIPCHCl3_, E**7**_2-MIPCHCl3_, T**7**_1-MIPCHCl3_ and T**7**_2-MIPCHCl3_ MIPs. The other systems were no longer pursued in subsequent studies.

#### 3.8.3. Time-Binding Study

To a set of triplicates of 30.0 mg of self-assembly systems E**7**_1-MIPCHCl3_, E**7**_2-MIPCHCl3_, T**7**_1-MIPCHCl3_ and T**7**_2-MIPCHCl3_, and semi-covalent MIPs E**16**_MIPCHCl3_ and T**16**_MIPCHCl3_ polymers, 1.00 mL of 0.0800 mM **1** was added and the mixture shaken for a designated time of contact. The binding times investigated were 0.5, 2.0, 4.0, 7.0 and 18 h. After binding, the mixtures were filtered, and the filtrates were analysed by HPLC. The amount of bound **1** was then obtained by subtracting the amount of **1** left in solution from the initial concentration.

#### 3.8.4. Saturation Binding

A series of 20.0 mg of self-assembly polymers E**7**_1-MIPCHCl3_, E**7**_2-MIPCHCl3_, T**7**_1-MIPCHCl3_ and T**7**_2-MIPCHCl3_ were incubated in different concentrations of **1** for 1 h, after which, the mixtures were filtered, and the filtrates were analysed directly by HPLC. The amount of bound **1** was then obtained by subtracting the amount of **1** left in solution from the initial concentration. Binding isotherms were generated from GraphPad Prism 9.4.1, and the best-fit values from non-linear regression, using the one-site–total-binding equation, were obtained.

The same procedure was followed for the semi-covalent MIPs E**16**_MIPCHCl3_ and T**16**_MIPCHCl3_, except that 30.0 mg of polymers was used for all binding measurements.

### 3.9. Selectivity Studies

For cross-reactivity tests, 20 mg of polymer was incubated with 1 mL of 0.8 mM analyte **1**, **17**, **18**, **19** or **20** in CHCl_3_ for 1 h. For binary competitive selectivity tests, 20 mg of polymer was incubated with 1 mL of 0.8 mM **1** mixed with 0.8 mM of **17**, **18** or **19** in CHCl_3_ for 1 h. The supernatant was analysed by either HPLC for **1**, **18**, **19** and **20** or GC–MS for **17**.

The HPLC method for **1**, **18** and **20** is outlined in Section 3.8.1. Analyte **17** was analysed using a Shimadzu 2010 gas chromatograph coupled to a Shimadzu QP2010 mass spectrometer and a Shimadzu AOC-20s auto sampler. The column was a ZB-5MS capillary column (30 m × 0.25 mm) coated with 0.25 μm of stationary phase. High-purity helium was used as the carrier gas at 71 kPa, with a column flow rate of 1 mL/min, a total flow rate of 9 mL/min and a split ratio of 15. Samples (1 μL) were injected and run using the following program: initial column temperature was 100 °C, which was held for 1 min before increasing to 300 °C at a rate of 10 °C/min. Analyte **19** was analysed using the following HPLC method: The mobile phase consisted of 75% aqueous buffer solution (50 mM K_2_HPO_4_ adjusted to pH 3.5 with H_3_PO_4_) and 25% 3:7 H_2_O:CH_3_CN (with 10 mM TEA). A 10 μL injection volume was used with a run time of 10 min, flow rate of 0.8 mL min^−1^ and detection wavelength of 190 nm. A calibration curve was generated using solutions in a range from 0.1 to 1 mM.

### 3.10. Physical Characterisation

#### 3.10.1. Scanning Electron Microscopy

Polymer morphology was examined using a Phillips XL30 scanning electron microscope. The sample was deposited on a sticky carbon tab and coated with gold, using a SPI gold spotter coating unit. SE micrographs of the polymers were obtained at 20,000× magnification at 15.0 kV.

#### 3.10.2. Swelling Measurements

A total of 30 (3) mg of each polymer was packed into an NMR tube, and the height of the dry polymer measured. A solution of **1** (1.00 mL of 0.0800 mM) in acetonitrile or chloroform was added and allowed to soak for 24 h. Polymers were allowed to settle, and the bed height of the swollen polymers was measured. The swelling factor was calculated from the ratio of the bed height of the swollen polymer to the dry polymer.

## 4. Conclusions

Using self-assembly (non-covalent) and semi-covalent methods, we designed **1**-specific MIPs. In the case of the self-assembly MIPs, a range of potential functional monomers (FM) were screened using a combination of pre-synthetic interaction studies (by molecular modelling and NMR analysis) and binding assays. The best performing self-assembly **1**-MIPs gave IFs of 3 to 7 and were formulated from FM **7** with ethylene glycol dimethacrylate (**E**GDMA) and trimethylolpropane trimethacrylate (**T**RIM) crosslinkers, using chloroform as porogen and rebinding solvent at T:FM ratios of 1:1 (E**7**_1-MIPCHCl3_ and T**7**_1-MIPCHCl3_) and 1:2 (E**7**_2-MIPCHCl3_ and T**7**_2-MIPCHCl3_). The binding parameters K_d_ and B_max_ were consistent with the MIPs exhibiting a stronger affinity towards **1** (lower K_d_), resulting in greater number of binding sites (higher B_max_) than their corresponding NIPs and observed to be higher for the 1:2 than the 1:1 T:FM formulations. The imprinting effect, as per IFs and NIP/MIP K_d_ ratios, was observed to be higher for the EGDMA-based MIPs than for the TRIM-based MIPs. The semi-covalent **1**-MIPs were designed using O-4-vinyl 4-benzylpiperazine-1-carbothioate or benzylpiperazine (4-vinylphenyl) carbamate (**16**) as the template–monomer (TM) adduct copolymerized with either EDGMA (E**16**_MIPCHCl3_) or TRIM (T**16**_MIPCHCl3_). We found that **1** could be cleaved from **16** post-polymerisation, leaving a phenol moiety within the imprinted sites capable of hydrogen bonding with **1** upon re-exposure. T**16**_MIPCHCl3_ exhibited a greater affinity for **1**—higher IFs, higher binding capacities and lower K_d_ than E**16**_MIPCHCl3_. The K_d_ values for semi-covalent MIPs are significantly lower than their self-assembly equivalents, while their B_max_ are, at the most, comparable.

The self-assembly MIPs displayed high levels of cross-reactivity with **19** and **20**, low to moderate with **18** and marginal with **17**. The analytes **17** and **18** are both large molecules that differ in shape, potential and available functional groups to **1** and are therefore a poor fit for the imprinted cavities. In contrast, **19** and **20** are similar in size and chemical character to **1**, consistent with the observed high cross-reactivity. The semi-covalent MIPs showed similar cross-reactivity trend to the self-assembly MIPs, except for analyte **17**, which recorded high levels of cross-reactivity. The unexpected high cross-reactivity with **17** could possibly be due to its benzoyl group, which is similar in structure and size to the benzyl group of **1**, and could easily fit the imprinted cavities and interact with the phenol moiety. Consistent with the results of the non-competitive cross-reactivity assays, the results of the competitive (binary mixtures) binding studies showed both self-assembly and semi-covalent MIPs to be highly selective towards **1** in the presence of **17**, moderately selective in the presence of **18** but non-selective in the presence of **19**. Competition of **1** with **20** was not studied due to separation problem.

The semi-covalent MIPs were observed to have a stronger affinity for **1** and faster uptake than the self-assembly systems. Both approaches gave MIPs of comparable binding selectivity and cross-reactivity. Overall, the observed IF values were significantly higher with the semi-covalent MIPs than with their self-assembly equivalents; however, it is worth noting that the semi-covalent NIP reference does not contain any FM, thus resulting in minimal **1** binding.

By virtue of its modest selectivity against the test for illicit drugs, **1**-MIP, could potentially be used as a dummy MIP for the broad-based capture and enrichment of illicit substances blended with **1,** e.g., **19** and substituted **20**, for subsequent laboratory analysis. Our preliminary data also demonstrated high affinity for **1** in an aqueous environment, thus raising the possibility for its use in illicit-drug testing.

## Figures and Tables

**Table 1:** Binding and thermodynamic parameters for E**7**_1-MIPCHCl3_, E**7**_2-MIPCHCl3_, T**7**_1-MIPCHCl3_ and T**7**_2-MIPCHCl3_ towards **1**.

Polymers		K_d_ (μM)	B_max_ (μmol/g)	K_d_ Ratio (NIP/MIP)	R^2^
**E7_1-MIPCHCl3_**	MIP	0.27 ± 0.07	56.2 ± 5.6	5.0	0.966
	NIP	1.36 ± 0.83	30.0 ± 10.4		0.884
**E7_2-MIPCHCl3_**	MIP	0.22 ± 0.04	111.7 ± 9.3	4.5	0.988
	NIP	0.98 ± 0.50	66.2 ± 19.3		0.909
**T7_1-MIPCHCl3_**	MIP	0.60 ± 0.16	45.9 ± 6.0	2.5	0.933
	NIP	1.52 ± 0.73	22.7 ± 6.3		0.934
**T7_2-MIPCHCl3_**	MIP	0.31 ± 0.07	90.1 ± 9.7	2.3	0.984
	NIP	0.71 ± 0.26	35.7 ± 7.2		0.937
**E16_MIPCHCl3_**	MIP	0.09 ± 0.02	16.8 ± 1.2	-	0.949
**T16_MIPCHCl3_**	MIP	0.02 ± 0.01	21.5 ± 1.7	-	0.869

K_d_ and B_max_ are the best-fit values at 95% confidence interval from non-linear regression of the binding isotherms (Supplementary Figure S19), using the one-site–total-binding equation generated using GraphPad Prism 9.4.1.

**Table 2:** Non-competitive cross-reactivity results for **1**-MIPs against cocaine (**17**), morphine (**18**), ephedrine (**19**) and phenylpiperazine (**20**).

MIP		1	17	18	19	20
**E7**_1-MIPCHCl3_	Bound (%) ^1^	51.5 ± 3.4	6.7 ± 0.8	17.4 ± 3.1	74.5 ± 5.8	52.1 ± 4.9
	ΔB (%) ^2^	33.5 ± 2.9	−1.3 ± 1.8	5.4 ± 3.8	34.8 ± 6.2	27.8 ± 5.5
	XRF ^3^	1.00	0.13	0.34	1.44	1.01
**E7**_2-MIPCHCl3_	Bound (%)	82.9 ± 2.2	12.2 ± 2.7	19.4 ± 7.4	82.9 ± 2.4	74.2 ± 3.9
	ΔB (%)	45.6 ± 2.3	2.8 ± 2.1	3.3 ± 3.1	23.4 ± 2.5	35.3 ± 2.2
	XRF	1.00	0.15	0.23	1.00	0.90
**T7**_1-MIPCHCl3_	Bound (%)	43.3 ± 3.4	3.5 ± 1.1	18.5 ± 2.5	54.9 ± 1.2	35.8 ± 4.0
	ΔB (%)	31.4 ± 3.6	0.6 ± 2.3	9.0 ± 2.6	24.6 ± 1.2	14.2 ± 4.0
	XRF	1.00	0.08	0.43	1.27	0.83
**T7**_2-MIPCHCl3_	Bound (%)	76.4 ± 3.4	11.2 ± 1.2	20.5 ± 4.2	76.8 ± 4.0	62.3 ± 1.8
	ΔB (%)	52.1 ± 5.0	2.2 ± 1.2	7.0 ± 4.5	25.1 ± 4.1	35.0 ± 4.0
	XRF	1.00	0.15	0.27	1.01	0.82
**E16**_MIPCHCl3_	Bound (%)	26.3 ± 2.5	16.8 ± 0.7	11.0 ± 2.0	26.0 ± 2.2	18.9 ± 1.5
	ΔB (%)	17.5 ± 3.2	8.9 ± 1.4	1.5± 2.3	12.1 ± 2.2	14.0 ± 2.2
	XRF	1.00	0.64	0.42	0.99	0.72
**T16**_MIPCHCl3_	Bound (%)	53.0 ± 1.4	45.0 ± 1.5	16.4 ± 2.2	39.8 ± 2.2	34.8 ± 4.4
	ΔB (%)	48.5 ± 1.9	41.0 ± 1.6	8.5 ± 2.3	36.8 ± 4.6	32.1 ± 4.5
	XRF	1.00	0.85	0.31	0.75	0.66

**^1^** Rebinding studies were conducted by incubating 20 mg of polymer in 1 mL of 0.8 mM solution of analyte in CHCl_3_ for 60 min. All binding tests were performed in triplicates. **^2^** ΔB = MIP binding − NIP binding. **^3^** XRF = cross-reactivity factor = analyte MIP binding/template **1** MIP binding.

**Table 3:** Binary competition results for **1**-MIPs against cocaine (**17**), morphine (**18**) and ephedrine (**19**).

Polymer	Binding ^1^	MIP				NIP			
		1	17	18	19	1	17	18	19
E**7**_1-MIPCHCl3_	Reference (%) ^2^	51.5 ± 3.4				18.0 ± 3.6			
	Normalised ^3^	1.09	0.19			1.19	0.41		
		1.05		0.38		0.77		0.38	
		0.94			1.73	0.66			2.50
	SI ^4^	1.00	0.18	0.37	1.85	1.00	0.34	0.49	3.80
E**7**_2-MIPCHCl3_	Reference (%)	82.9 ± 2.2				37.2 ± 3.1			
	Normalised	0.93	0.15			1.11	0.16		
		0.86		0.22		0.88		0.02	
		0.86			1.06	0.90			1.89
	SI	1.00	0.16	0.26	1.23	1.00	0.14	0.02	2.11
T**7**_1-MIPCHCl3_	Reference (%)	43.3 ± 3.4				11.9 ± 1.2			
	Normalised	1.12	0.18			1.90	0.56		
		1.24		0.20		1.64		0.56	
		0.74			1.29	1.15			3.15
	SI	1.00	0.16	0.16	1.74	1.00	0.30	0.34	2.74
T**7**_2-MIPCHCl3_	Reference (%)	76.4 ± 3.4				24.3 ± 3.6			
	Normalised	0.89	0.11			1.11	0.10		
		0.89		0.20		1.38		0.26	
		0.78			1.04	0.92			2.28
	SI	1.00	0.12	0.23	1.32	1.00	0.09	0.19	2.47
E**16**_MIPCHCl3_	Reference (%)	26.3 ± 2.5				8.7 ± 2.0			
	Normalised	1.01	0.07			0.72	0.37		
		0.33		0.15		0.35		0.20	
		0.63			1.14	0.17			1.54
	SI	1.00	0.07	0.46	1.81	1.00	0.51	0.59	8.85
T**16**_MIPCHCl3_	Reference (%)	53.0 ± 1.4				4.5 ± 1.3			
	Normalised	0.71	0.13			0.89	0.60		
		0.33		0.20		0.63		0.66	
		0.42			0.57	0.34			1.18
	SI	1.00	0.18	0.61	1.34	1.00	0.68	1.05	3.50

**^1^** Rebinding studies were conducted by incubating 20 mg of polymer in 1 mL of 0.8 mM solution of analyte in CHCl_3_ for 60 min. All binding tests were performed in triplicates. **^2^** Binding of **1** as sole analyte. **^3^** Binding of binary analytes with respect to reference. **^4^** SI = selectivity index = normalised analyte binding/normalised **1** binding.

## Data Availability

Not applicable.

## Supplementary Materials

The supporting information can be downloaded at https://www.mdpi.com/article/10.3390/ijms24065117/s1.
