# Supplementary material for: Molecular Imprinting of Benzylpiperazine: A Comparison of the Self-Assembly and Semi-Covalent Approaches
Source: Int J Mol Sci. 2023 Mar 7;24(6):5117. doi: 10.3390/ijms24065117 (PMC10048891; doi:10.3390/ijms24065117)
Supplement: Supplementary file 1 [file ijms-24-05117-s001.zip › ijms-2200174-supplementary.pdf]

Molecular imprinting of benzylpiperazine: a comparison of the self-assembly and semi-covalent approaches

Kathleen M. Wright [1,2], Michael C. Bowyer [3], Adam McCluskey [2] and Clovia I. Holdsworth [2,*]

[1] Sanitarium Health Food Company, 1 Sanitarium Drive, Berkeley Vale, NSW 2261, Australia
[2] School of Environmental and Life Sciences, University of Newcastle, Callaghan, NSW 2308, Australia
[3] Academic Division, University of Newcastle, Ourimbah, NSW 2258, Australia
* Correspondence: clovia.holdsworth@newcastle.edu.au; Tel.: +61-2-4921-5481

Electronic Supporting Information

Contents

1. **Figures S1-S5**. Molecular modelling images of the energy minimised interactions between selected functional monomers and benzylpiperazine.

2. **Figures S6-S8**. Molecular modelling images of the energy minimised interactions between cross linkers and benzylpiperazine.

3. **Figures S9-S13**. NMR titration experiments with benzylpiperazine and selected functional monomers (acrylic acid, methacrylic acid, itaconic acid and 7-hydroxy-4-methylcoumarin acrylate).

4. **Figures S14-S15**. NMR titration experiments with benzylpiperazine and crosslinkers NOBE, EGDMA, TRIM and DVB.

5. **Figures S16-S18**. SEM images of self-assembly MIPs and NIPs.

6. **Figure S19**. Binding isotherms produced for (**A**) E**7**$_{1\text{-MIPCHCl3}}$; (**B**) E**7**$_{2\text{-MIPCHCl3}}$; (**C**) T**7**$_{1\text{-MIPCHCl3}}$; and (**D**) T**7**$_{2\text{-MIPCHCl3}}$ and (**E**) semi-covalent E**16**$_{\text{MIPCHCl3}}$ and T**16**$_{\text{MIPCHCl3}}$.

7. **Figure S20**. SEM images for (A) E**16**$_{\text{MIPCHCl3}}$, (B) E$_{\text{NIP}}$, (C) T**16**$_{\text{MIPCHCl3}}$ and (D) T$_{\text{NIP}}$.

8. **Figure S21**. Cross-reactivity data for (**A**) E**7**$_{1\text{-MIPCHCl3}}$; (**B**) E**7**$_{2\text{-MIPCHCl3}}$; (**C**) T**7**$_{1\text{-MIPCHCl3}}$; (**D**) T**7**$_{2\text{-MIPCHCl3}}$; (**E**) E**16**$_{\text{MIPCHCl3}}$; and (**F**) T**16**$_{\text{MIPCHCl3}}$.

9. **Figure S22-S23**. Molecular modelling images of **1**, **17**, **18**, **19** and **20** showing their surface potentials and sizes.

10. **Figure S24**. Competitive binary binding data for (**A**) E**7**$_{1\text{-MIPCHCl3}}$; (**B**) E**7**$_{2\text{-MIPCHCl3}}$; (**C**) T**7**$_{1\text{-MIPCHCl3}}$; (**D**) T**7**$_{2\text{-MIPCHCl3}}$; (**E**) E**16**$_{\text{MIPCHCl3}}$; and (**F**) T**16**$_{\text{MIPCHCl3}}$.

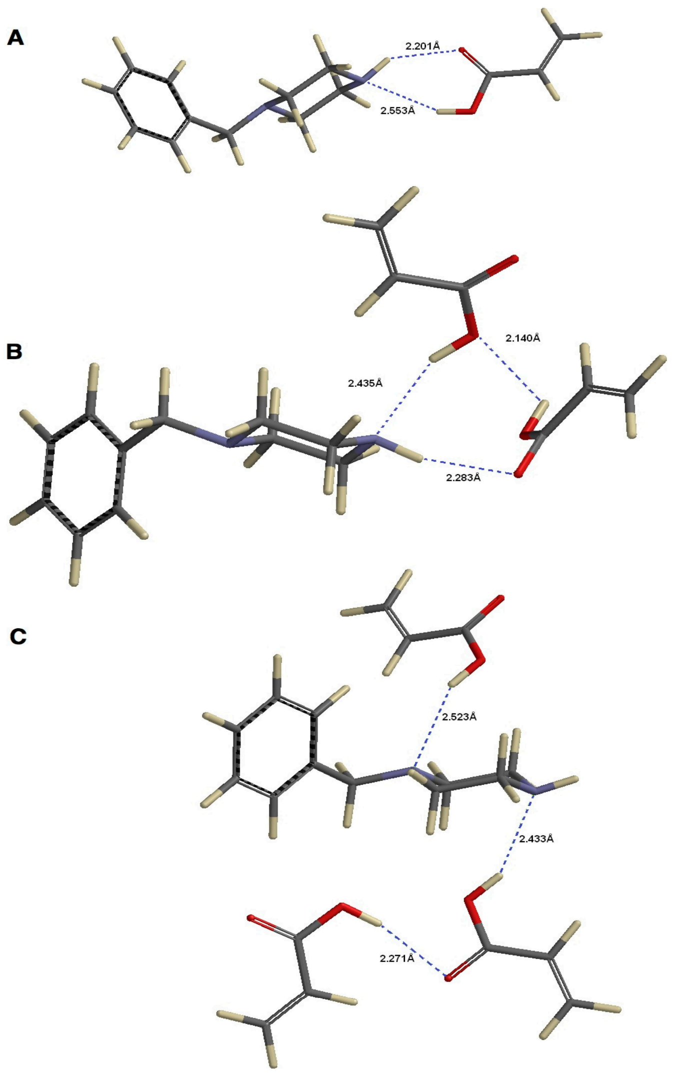

Figure S1. Energy minimised computer modelling representations of the interaction of benzylpiperazine (**1**) with acrylic acid (**6**) in the ratios, **A**) 1:1; **B**) 1:2; and **C**) 1:3.

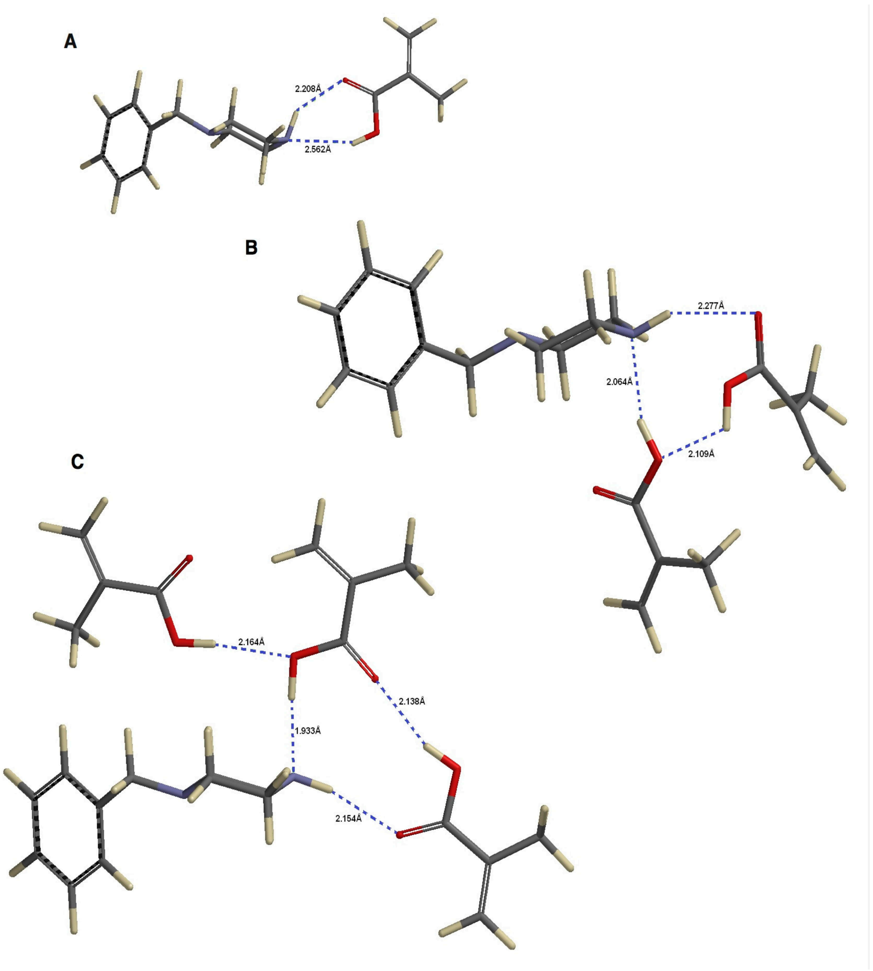

Figure S2. Energy minimised computer modelling representations of the interaction of benzylpiperazine (**1**) with methacrylic acid (**7**) in the ratios of, **A**) 1:1; **B**) 1:2; and **C**) 1:3.

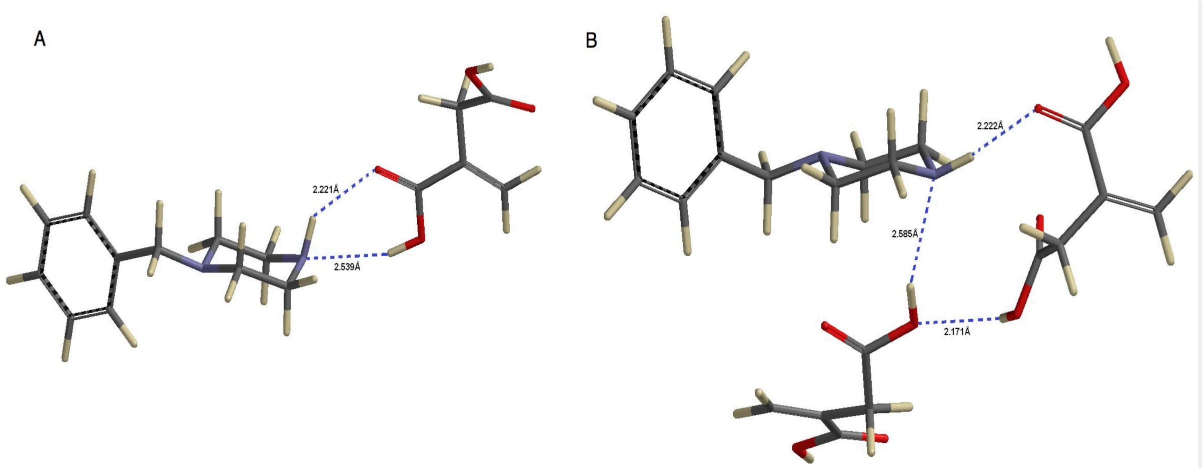

Figure S3. Energy minimised computer modelling representations of the interaction of benzylpiperazine (**1**) with itaconic acid (**12**) in the ratios of, **A**) 1:1; and **B**) 1:2.

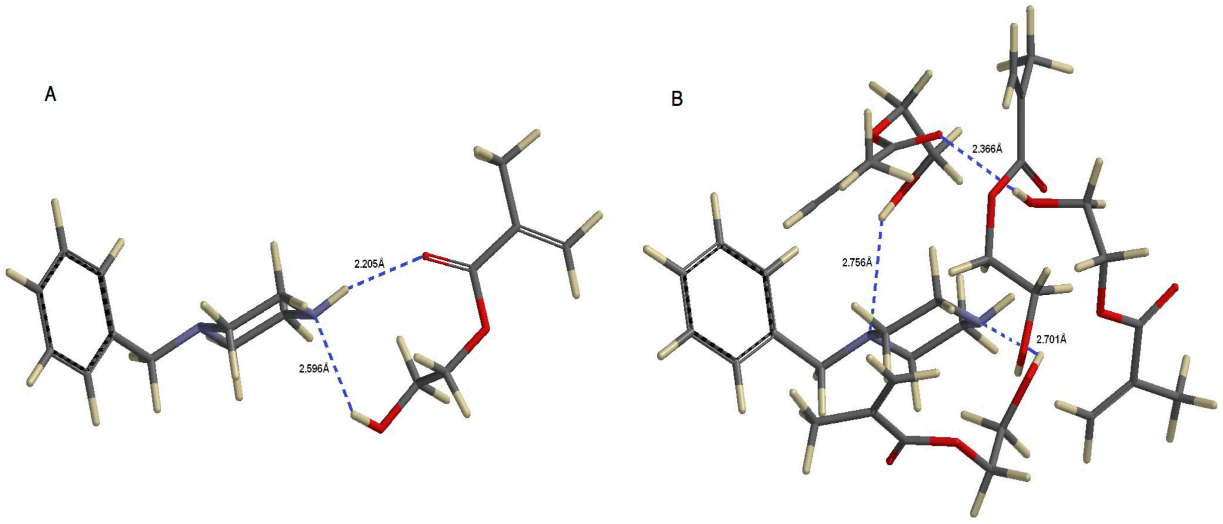

Figure S4. Energy minimised computer modelling representations of the interaction of benzylpiperazine (**1**) with 2-hydroxymethyl methacrylate (**13**) in the ratios of, **A**) 1:1 and **B**) 1:4.

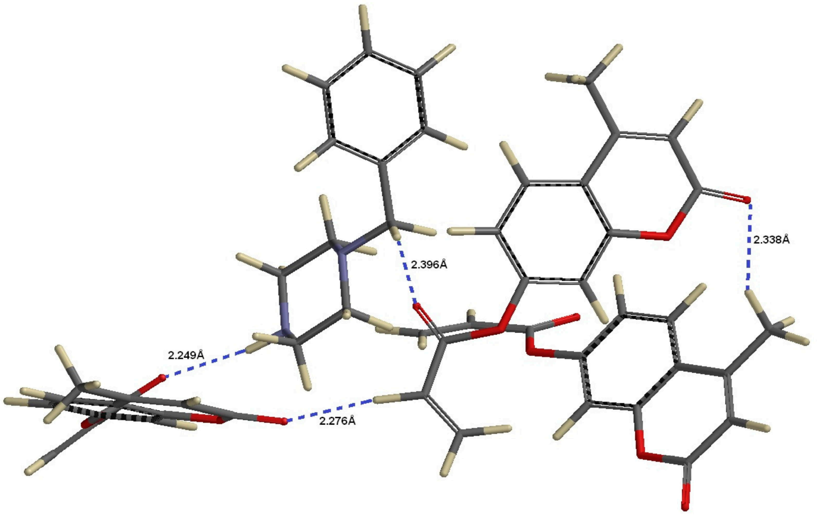

Figure S5. Energy minimised computer modelling representations of the interaction of benzylpiperazine (**1**) with 7-hydroxy-4-methylcoumarin acrylate (**15**) in the ratio of 1:3.

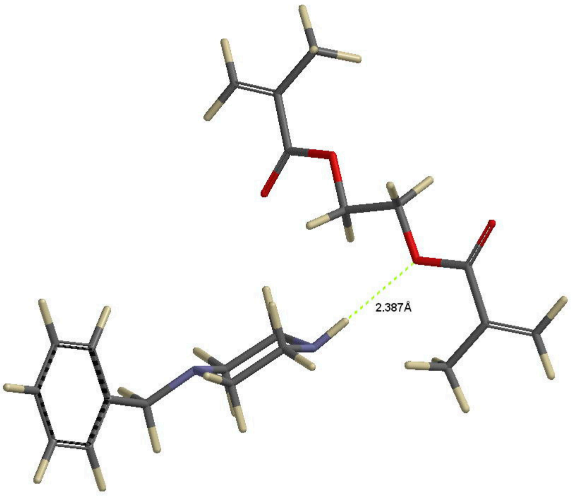

Figure S6. Energy minimised computer modelling representations of the interaction of benzylpiperazine (**1**) with EGDMA in an optimised 1:1 ratio.

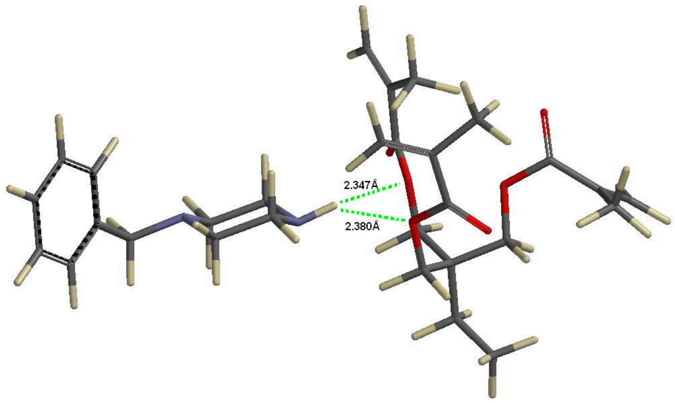

Figure S7. Energy minimised computer modelling representations of the interaction of benzylpiperazine (**1**) with TRIM in an optimised 1:1 ratio.

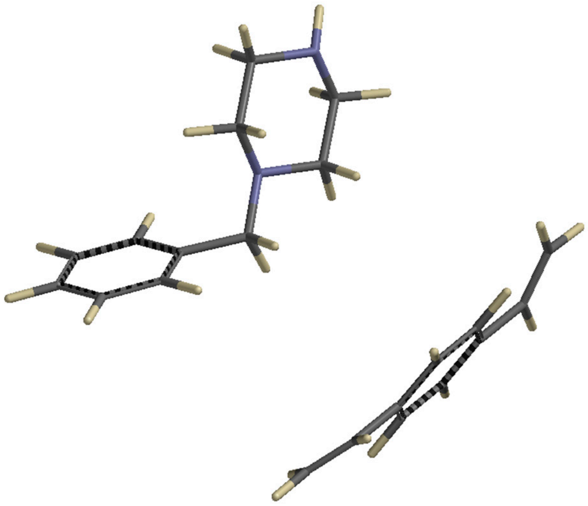

Figure S8. Computer generated molecular modelling image for benzylpiperazine (**1**) with divinylbenzene (DVB) for the geometry optimised 1:1 cluster.

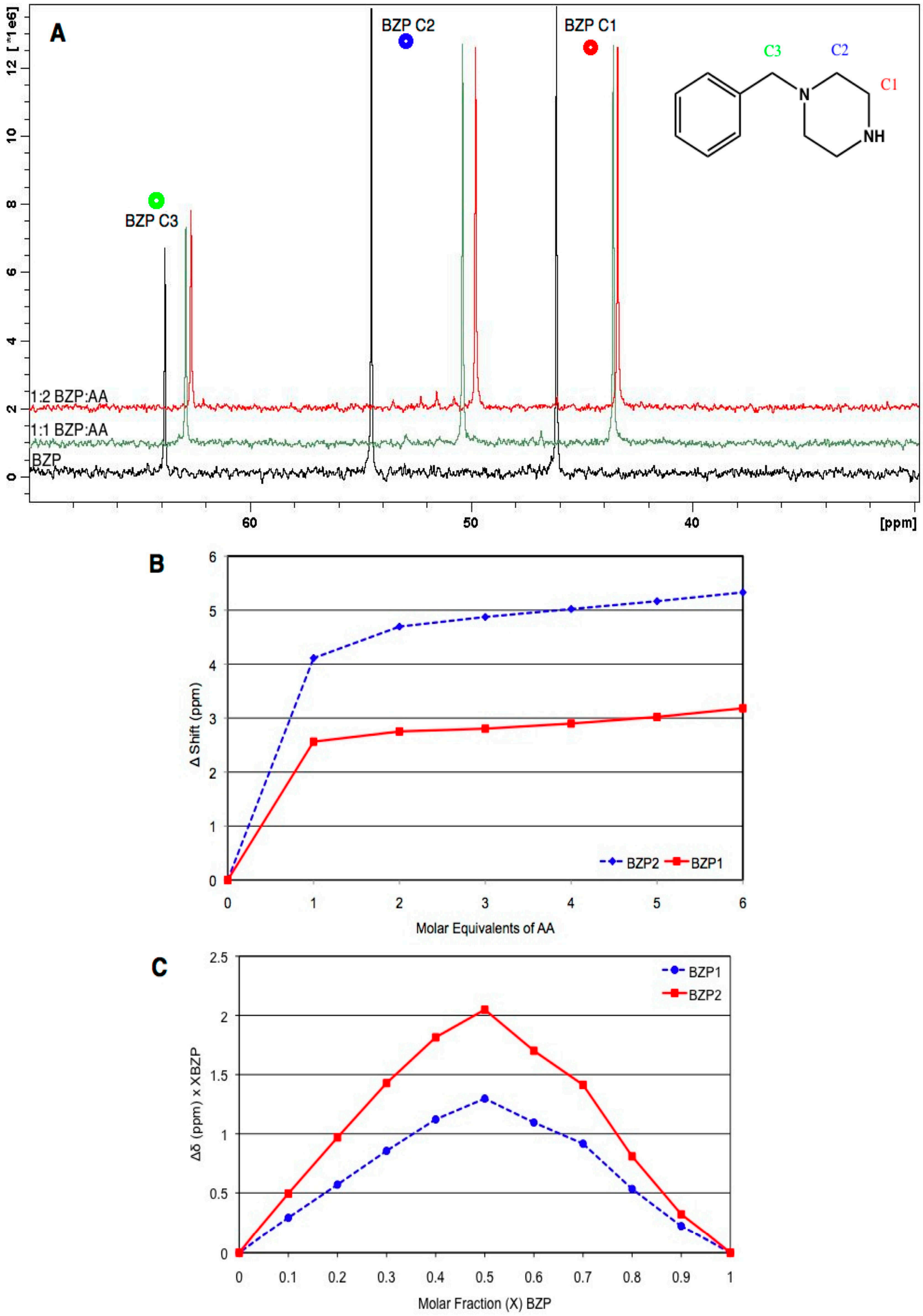

Figure S9. NMR analysis of benzylpiperazine (**BZP**, **1**) with acrylic acid (**AA**, **6**) showing, **A**) the ^{13}C NMR spectra for the NMR titration of **BZP** with **AA** at a T:FM ratio of 1:0, 1:1 and 1:2; **B**) the changes in chemical shift of carbons 1 and 2 on benzylpiperazine (**1**) as a function of increasing **6** concentration; and **C**) Job's plot of two selected **BZP** carbon resonances (C1 and C2) in the presence of **AA**. Cluster stoichiometry is 1:1. ^{13}C NMR spectra obtained at 28 ºC in deuterated chloroform.

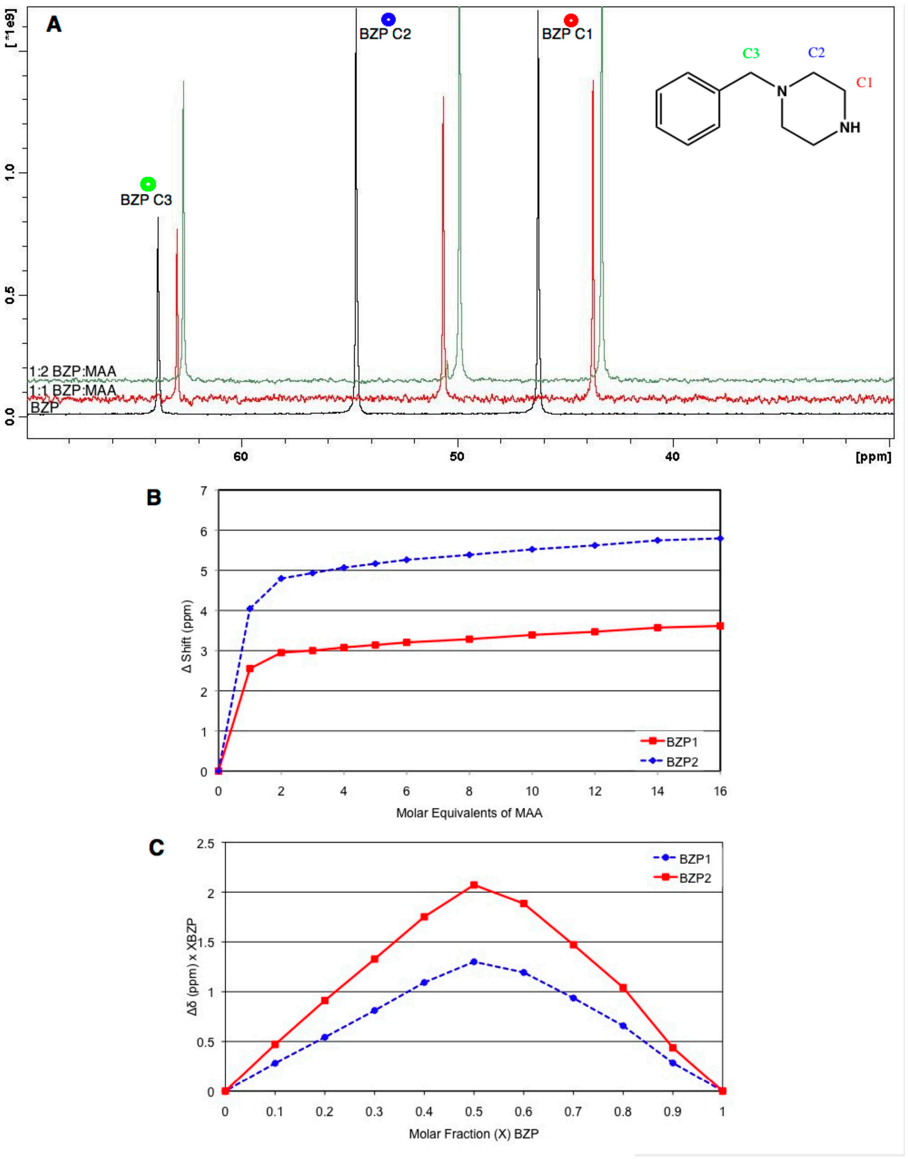

Figure S10. NMR analysis of benzylpiperazine (**BZP**, **1**) with methacrylic acids (**MMA**, **7)** showing, **A**) the ^{13}C NMR spectra for the NMR titration of **BZP** (**1**) with **MAA** (**7**) at a T:FM ratio of 1:0 (no **7** added), 1:1 and 1:2; **B**) the changes in chemical shift of **BZP** carbons 1 and 2 as a function of increasing **MAA** (**7**) concentration; and **C**) Job's plot of two selected **BZP** carbon s (C1 and C2) in the presence of **MAA** showing the predominant cluster stoichiometry as 1:1. ^{13}C NMR spectra obtained at 28 ºC in deuterated chloroform.

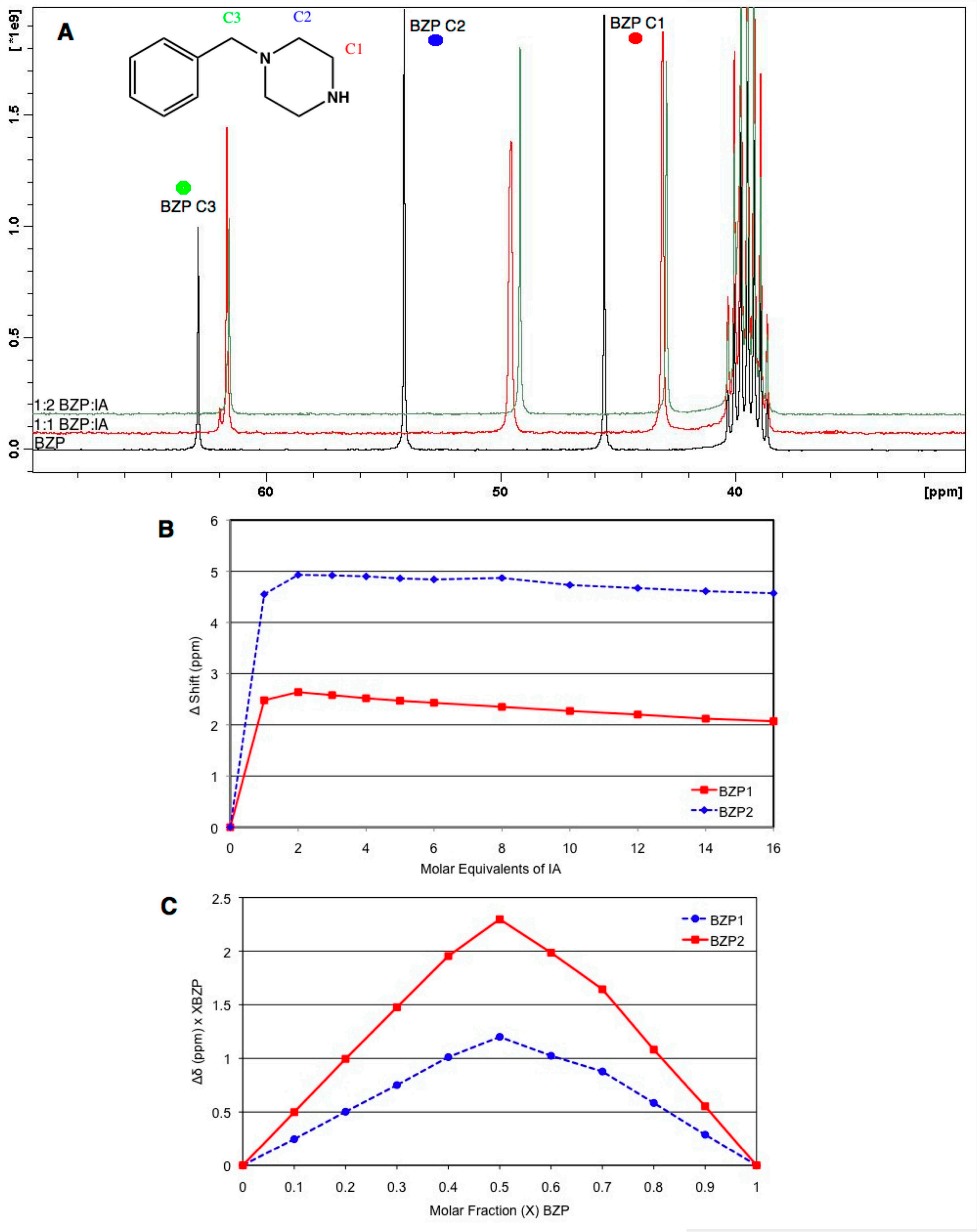

Figure S11. NMR analysis of benzylpiperazine (**BZP**, **1**) with itaconic acid (**IA**, **12**) showing, **A**) the ^{13}C NMR spectra for the NMR titration of BZP (**1**) with IA at a T:FM ratio of 1:0, 1:1 and 1:2 BZP (**1**):IA; **B**) the changes in chemical shift of carbons 1 and 2 on **BZP** as a function of increasing **IA** concentration; and **C**) Job's plot of two selected **BZP** carbon resonances (C1and C2) in the presence of **IA**. Cluster stoichiometry is 1:1. ^{13}C NMR spectra obtained at 28 ºC in deuterated DMSO.

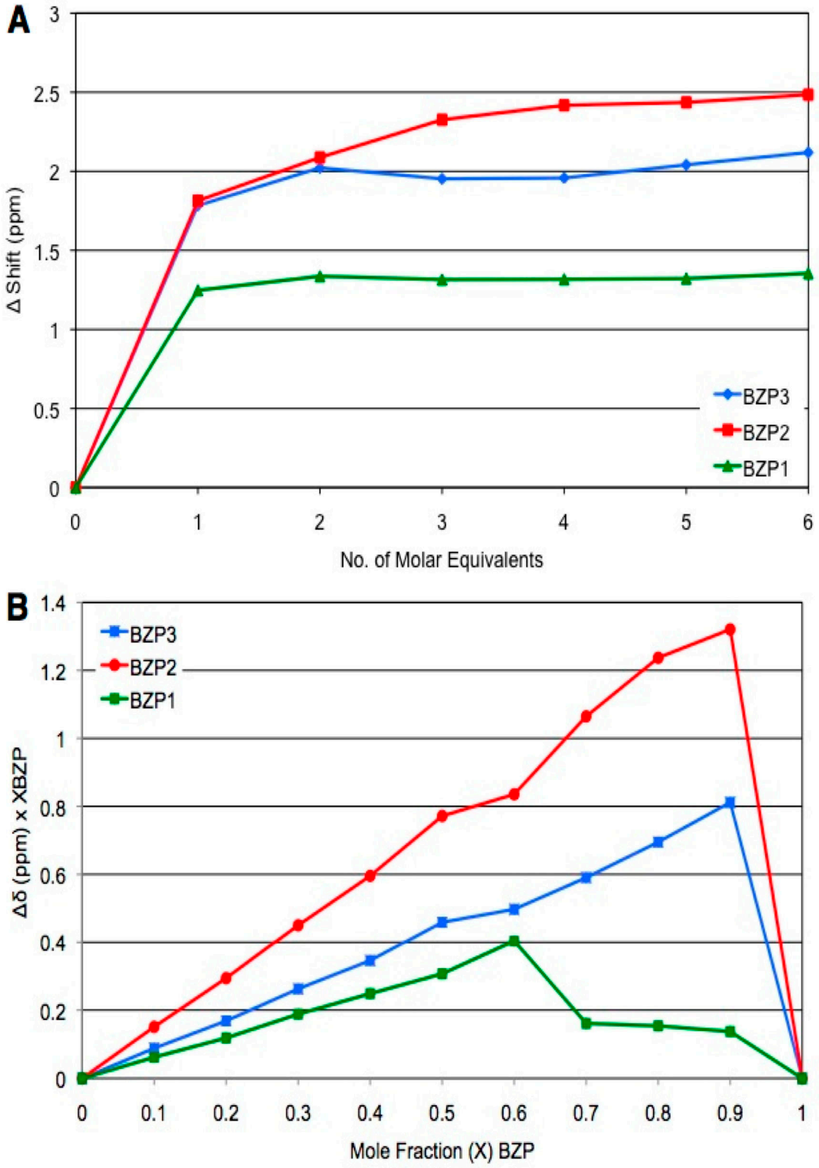

Figure S12. NMR analysis of benzylpiperazine (**BZP**, **1**) with 7-hydroxy-4-methylcoumarin acrylate (**HMCA**, **15**) showing, **A**) the changes in chemical shift of carbons 1 and 2 on **BZP** as a function of increasing **HMCA** concentration; and **B**) Job's plot of two selected benzylpiperazine (**1**) carbon resonances (C1 and C2) in the presence of HMCA. Cluster stoichiometry was 9:1 **BZP**:**HMCA**. ^{13}C NMR spectra obtained at 28 °C in deuterated chloroform.

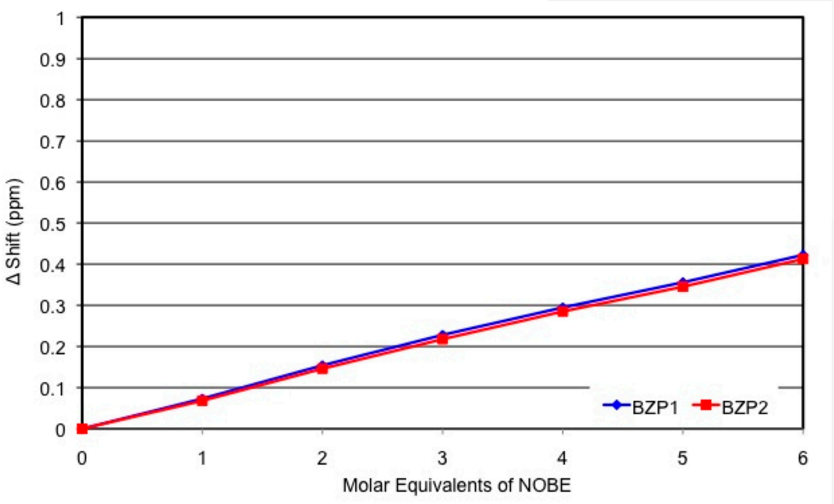

Figure S13. NMR titration of benzylpiperazine (**BZP**, **1**) with N,O-bismethacroyloyl ethanolamine (**NOBE**, **14**) showing the changes in chemical shift of carbons 1 and 2 on **BZP** as a function of increasing **NOBE** concentration.

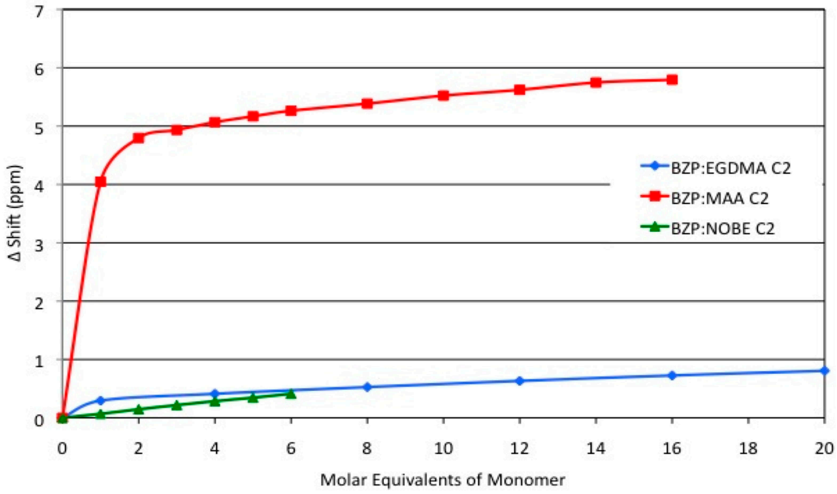

Figure S14. NMR titration of benzylpiperazine (**BZP**, **1**) with N,O-bismethacroyloyl ethanolamine (**NOBE**, **14**), methacrylic acid (**MAA**, **7**) and ethylene glycol dimethylacrylate (**EGDMA**) showing the changes in chemical shift of carbon 1 on **BZP** as a function of increasing monomer concentration.

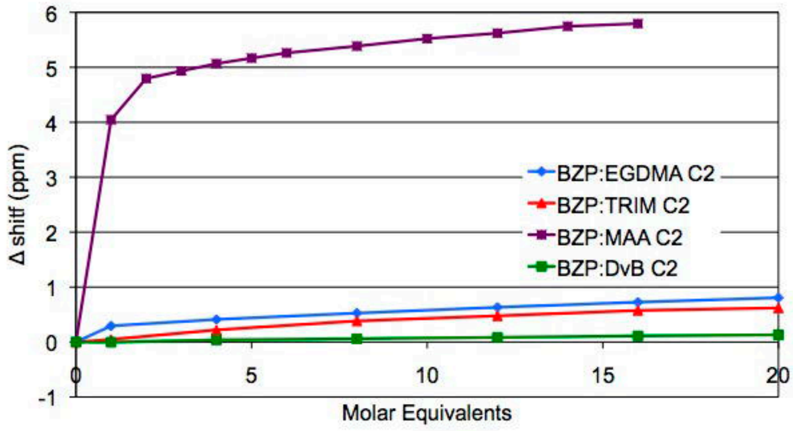

Figure S15. NMR titration of benzylpiperazine (**BZP**, **1**) with ethylene glycol dimethylacrylate (**EGDMA**), trimethylolpropane trimethylacrylate (**TRIM**), divinylbenzene (**DVB**) and methacrylic acid (**MAA**, **7**) showing the most significant change in chemical shift experienced as a function of increasing **EGDMA**, **TRIM**, **DVB** and **MAA** concentration, respectively. **MAA** has been included for comparison

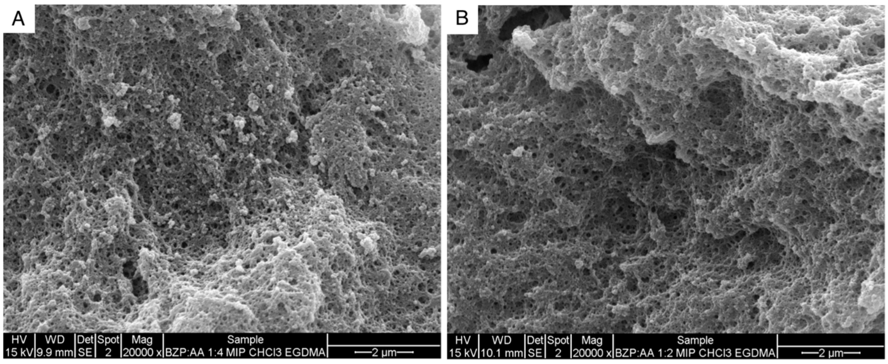

Figure S16. SEM images for A) E**6**$_4$-MIP$_{CHCl3}$ and B) E**6**$_2$-MIP$_{CHCl3}$ MIPs obtained at 20000x magnification at 15.0 kV.

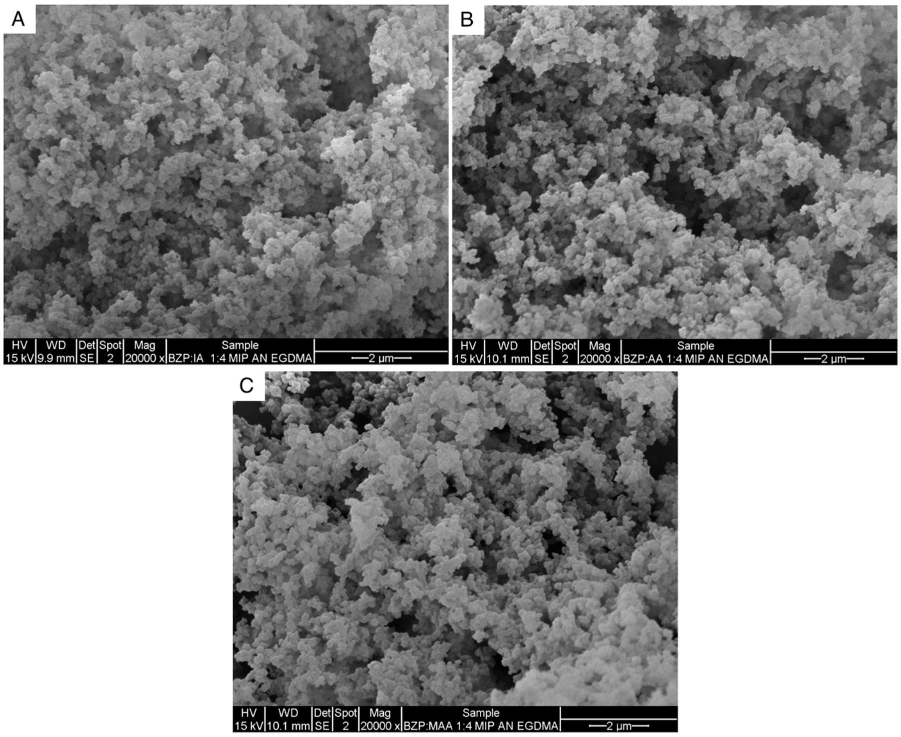

Figure S17. SEM images for A) E**12**$_4$-MIP$_{CH3CN}$, B) E**6**$_4$-MIP$_{CH3CN}$ and C) E**7**$_4$-MIP$_{CH3CN}$ MIPs obtained at 20000x magnification at 15.0 kV.

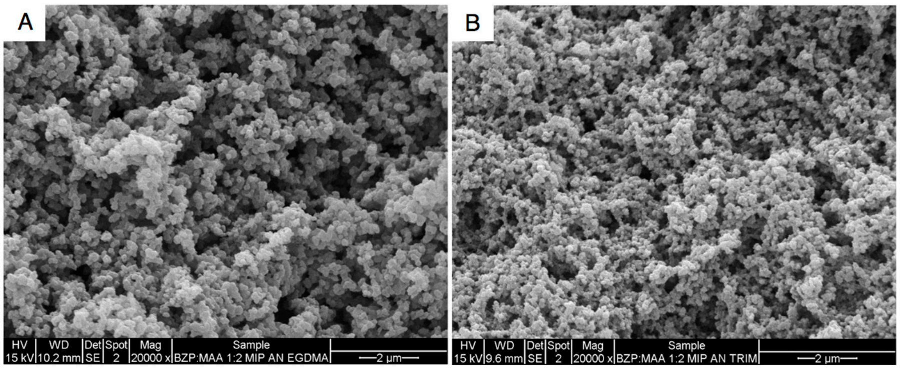

Figure S18. SEM images for A) E**7**$_2$-MIP$_{CH3CN}$, and B) T**7**$_2$-MIP$_{CH3CN}$ MIPs obtained at 20000x magnification at 15.0 kV.

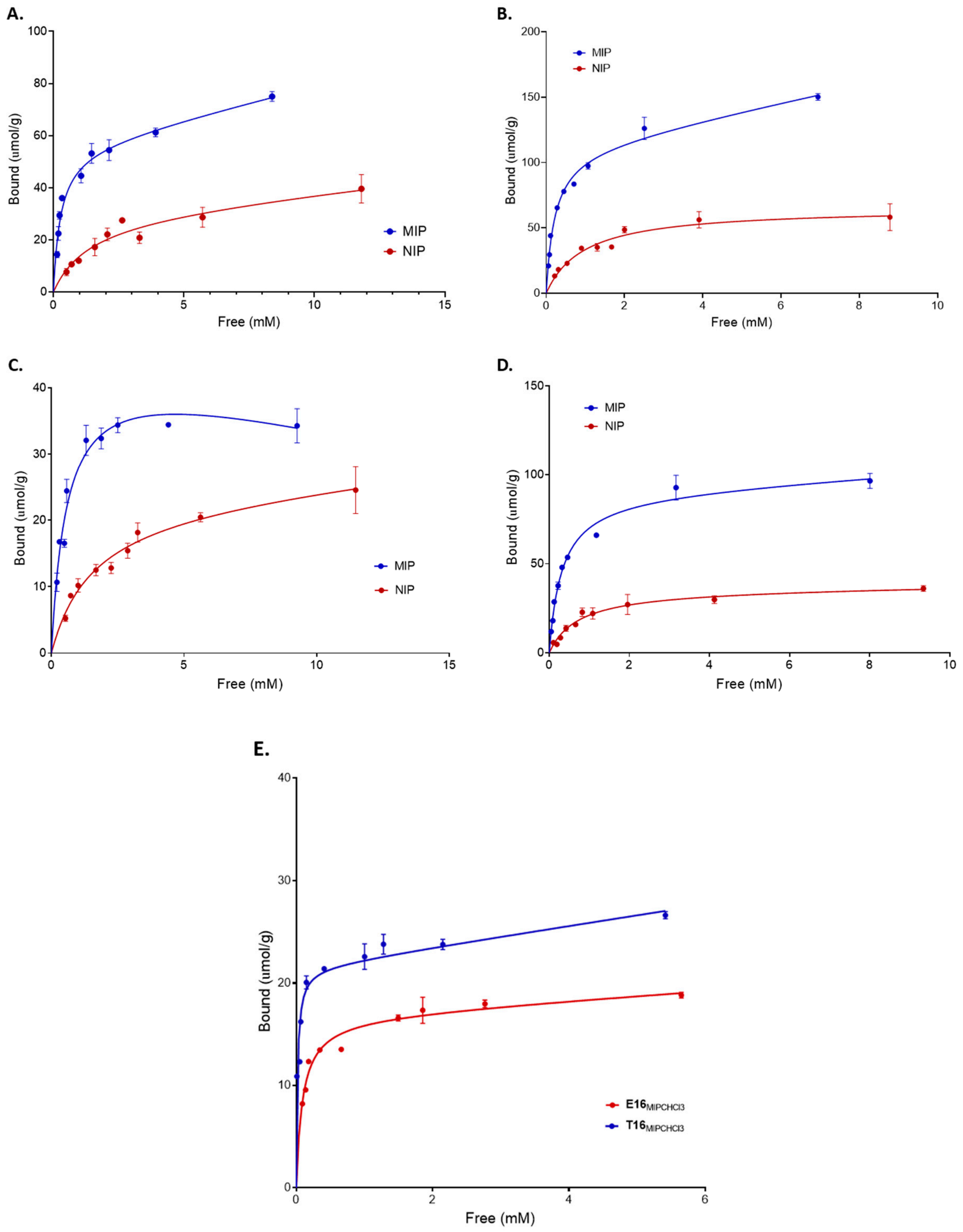

Figure S19. Binding isotherms produced for (**A**) E**7**$_{1\text{-MIPCHCl3}}$; (**B**) E**7**$_{2\text{-MIPCHCl3}}$; (**C**) T**7**$_{1\text{-MIPCHCl3}}$; and (**D**) T**7**$_{2\text{-MIPCHCl3}}$ and (**E**) semi-covalent E**16**$_{\text{MIPCHCl3}}$ and T**16**$_{\text{MIPCHCl3}}$.

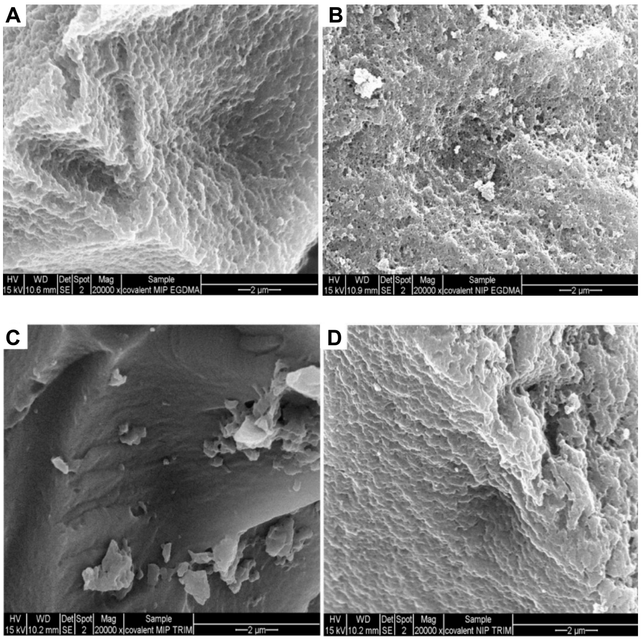

Figure S20. SEM images for (**A**) E**16**$_{MIPCHCl3}$, (**B**) E_{NIP}, (**C**) T**16**$_{MIPCHCl3}$ and (**D**) T_{NIP}.

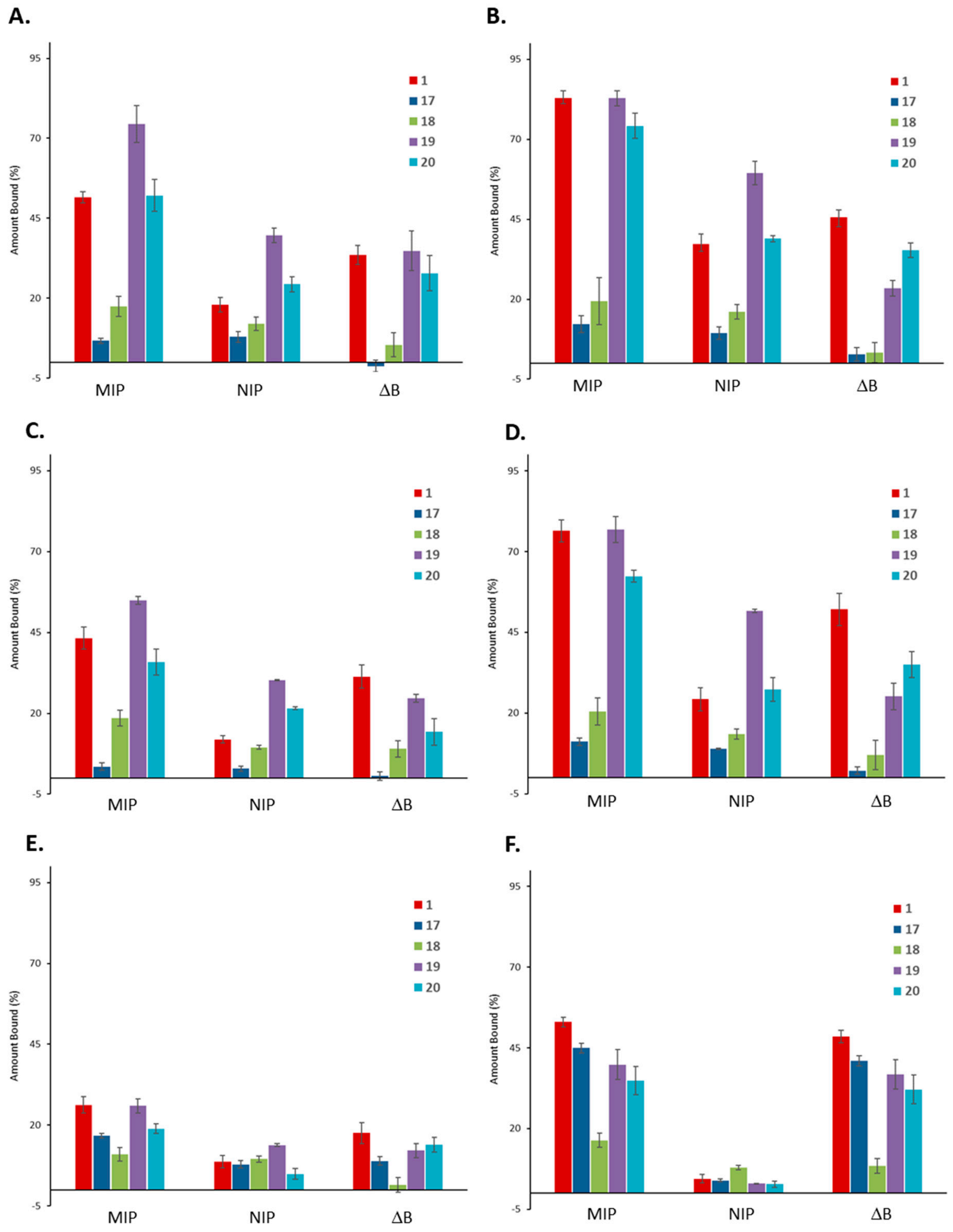

Figure S21. Cross-reactivity data for (**A**) E**7**$_{1\text{-}MIPCHCl3}$; (**B**) E**7**$_{2\text{-}MIPCHCl3}$; (**C**) T**7**$_{1\text{-}MIPCHCl3}$; (**D**) T**7**$_{2\text{-}MIPCHCl3}$; (**E**) E**16**$_{MIPCHCl3}$; and (**F**) T**16**$_{MIPCHCl3}$ using 20 mg of polymer and 0.8 mM analyte solutions at t = 60 min in triplicate.

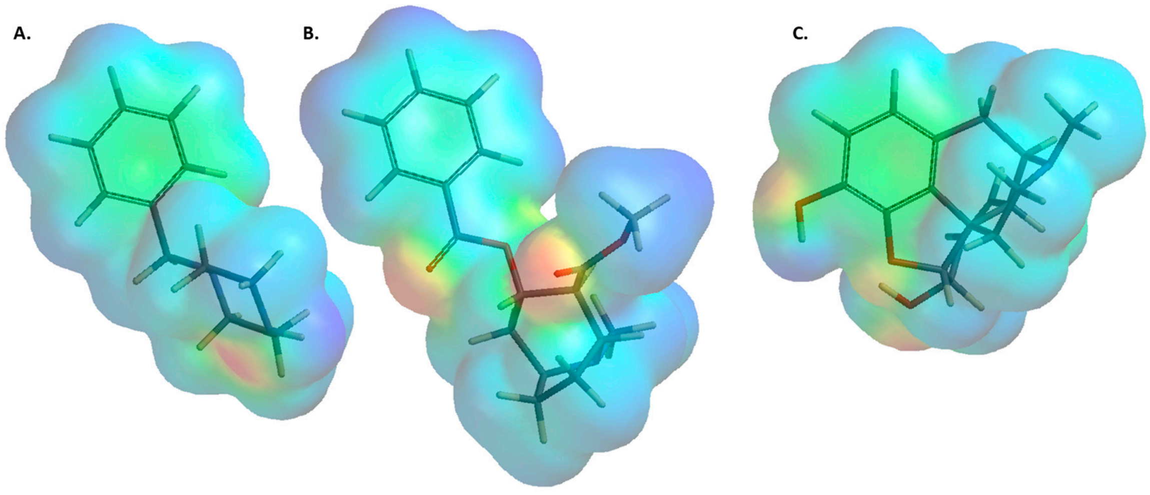

Figure S22. Molecular modelling images of **1** (**A**), **17** (**B**) and **18** (**C**) showing their differences in surface potential and size.

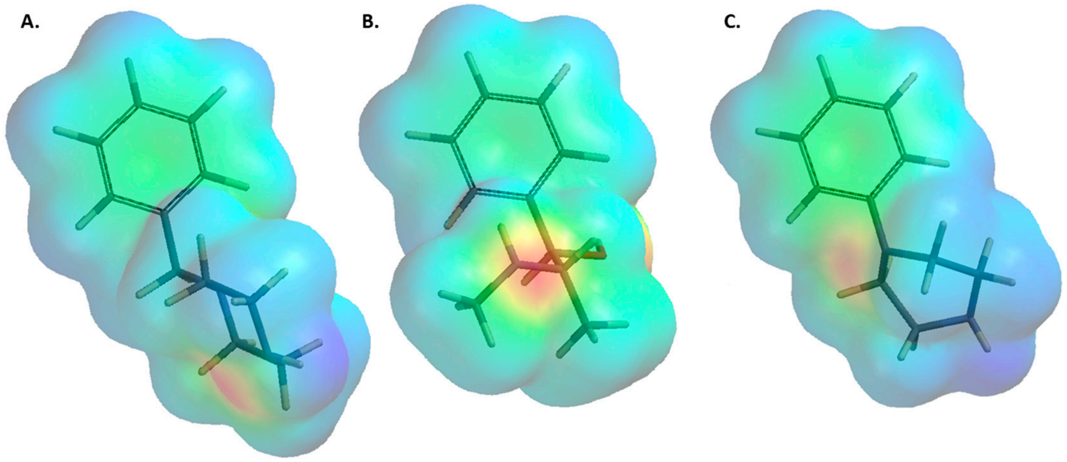

Figure S23. Molecular modelling images of **1** (**A**), **19** (**B**) and **20** (**C**) showing their similarities in surface potential and size.

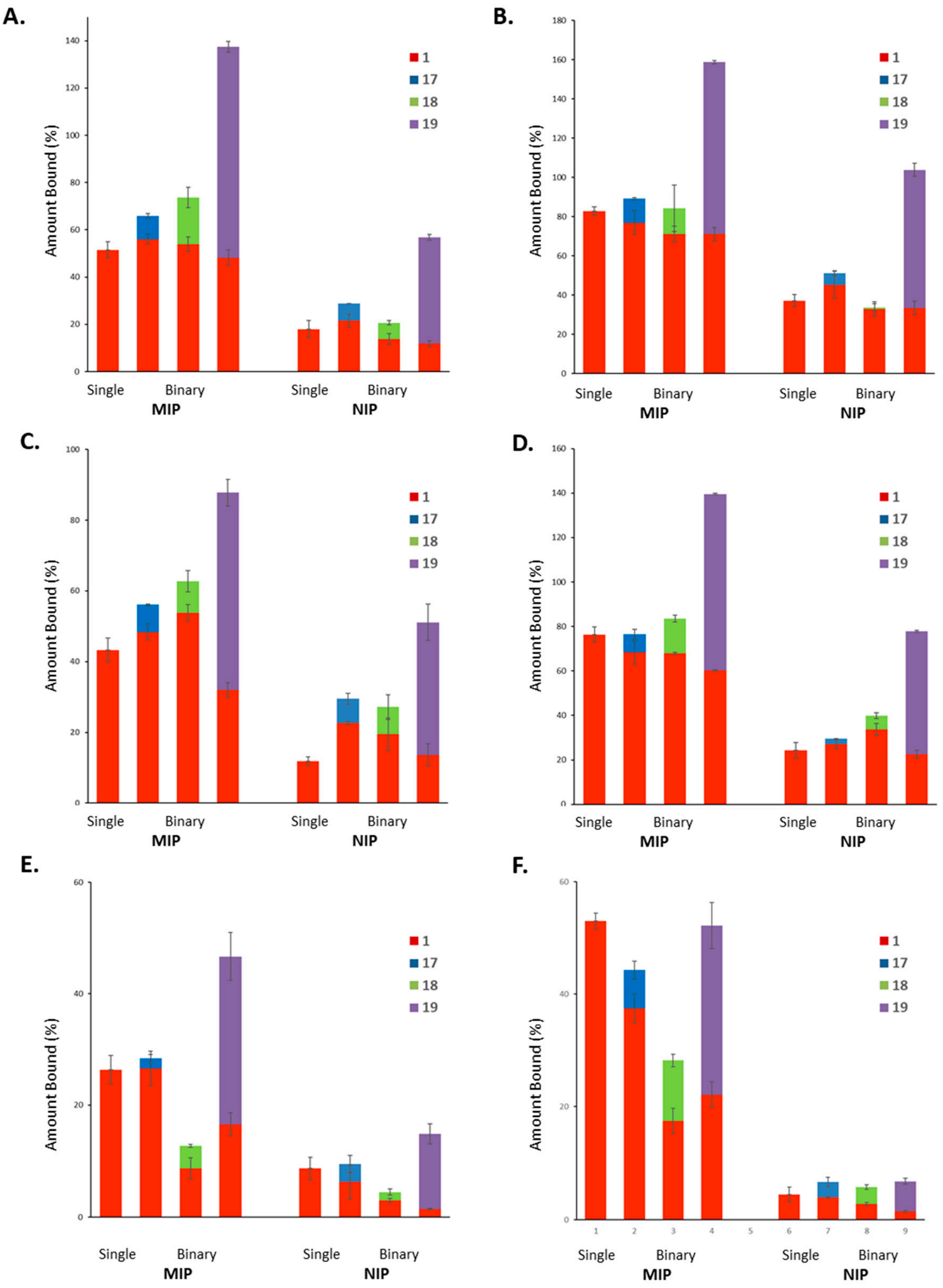

Figure S24. Competitive binary binding data for (**A**) E**7**$_{1\text{-MIPCHCl3}}$; (**B**) E**7**$_{2\text{-MIPCHCl3}}$; (**C**) T**7**$_{1\text{-MIPCHCl3}}$; (**D**) T**7**$_{2\text{-MIPCHCl3}}$; (**E**) E**16**$_{\text{MIPCHCl3}}$; and (**F**) T**16**$_{\text{MIPCHCl3}}$ using 20 mg of polymer and a mixture of 0.8 mM solution of **1** and 0.8 mM solution of analyte (**17**, **18** or **19**) at t = 60 min in triplicate. Binding is expressed in % with respect to the original concentration; for a binary system, this would translate to values higher than 100%.
